# SDImpute: A statistical block imputation method based on cell-level and gene-level information for dropouts in single-cell RNA-seq data

**DOI:** 10.1371/journal.pcbi.1009118

**Published:** 2021-06-17

**Authors:** Jing Qi, Yang Zhou, Zicen Zhao, Shuilin Jin

**Affiliations:** School of Mathematics, Harbin Institute of Technology, Harbin, P.R, China; University of California Los Angeles, UNITED STATES

## Abstract

The single-cell RNA sequencing (scRNA-seq) technologies obtain gene expression at single-cell resolution and provide a tool for exploring cell heterogeneity and cell types. As the low amount of extracted mRNA copies per cell, scRNA-seq data exhibit a large number of dropouts, which hinders the downstream analysis of the scRNA-seq data. We propose a statistical method, SDImpute (Single-cell RNA-seq Dropout Imputation), to implement block imputation for dropout events in scRNA-seq data. SDImpute automatically identifies the dropout events based on the gene expression levels and the variations of gene expression across similar cells and similar genes, and it implements block imputation for dropouts by utilizing gene expression unaffected by dropouts from similar cells. In the experiments, the results of the simulated datasets and real datasets suggest that SDImpute is an effective tool to recover the data and preserve the heterogeneity of gene expression across cells. Compared with the state-of-the-art imputation methods, SDImpute improves the accuracy of the downstream analysis including clustering, visualization, and differential expression analysis.

## Author summary

Single-cell RNA sequencing (scRNA-seq) allows researchers to analyze gene expression of thousands of single cells simultaneously. However, the low amount of extracted mRNA leads to a large number of dropout events, which introduce computational challenges and hinder downstream analysis of data. To address this problem, we developed SDImpute, a novel statistical method to recover the scRNA-seq data based on cell-level and gene-level information in this manuscript. The goal of our algorithm is to denoise the scRNA-seq data while maintaining the biological nature of gene expression. Combining SDImpute with the downstream analysis tools, we considered the matched bulk expression data and known cell labels of the scRNA-seq data as criteria to design experiments to validate the performance of our method in both simulated and real datasets. Moreover, we offer an R package with detailed instructions and an example input dataset. We hope that SDImpute will be beneficial to researchers to identify mechanisms underlying some biological processes by analysis of the scRNA-seq data.

This is a *PLOS Computational Biology* Methods paper.

## Introduction

The scRNA-seq technologies quantify the heterogeneity of cell transcriptomes at a high resolution and discover novel cell types, which is superiority over bulk RNA-seq technologies [1–5]. However, due to the low amounts of extracted mRNA from cells, the scRNA-seq data is generally mixed with technical noise and much more zero counts in the expression matrix than the bulk RNA-seq data. The excess zero counts in the scRNA-seq data are called “dropout” [6–8]. In the scRNA-seq dataset, it is not uncommon to have over 50% of expressions in the count matrix equal to zero [9–10]. Therefore, it is a severe computational challenge to impute the dropout events, which greatly influence the accuracy of the downstream analysis [10–14].

Until now, several methods were designed for dealing with the dropout events in the scRNA-seq data [15–23]. These methods capture dropout features in different ways and implement imputation strategies by borrowing information from similar cells or similar genes. Some methods rely on the cell-level information (the information comes from the other similar cells) to impute dropouts [16–19]. For instance, MAGIC constructs an affinity matrix to impute dropouts by sharing information across similar cells based on the theory of heat diffusion geometry [16]. DrImpute finds similar cells by clustering repeatedly and imputes missing values by averaging the gene expression values from similar cells and then averages the multiple estimations as to the final imputation value [17]. VIPER imputes the missing values by borrowing information across local neighborhood cells based on a non-negative sparse regression model [18]. Besides, scImpute identifies the dropout events based on the Gamma-Normal mixture model and imputes dropouts by borrowing information from similar cells using non-negative least squares regression [19]. Other methods infer the imputed value using the gene-level information (the information comes from the other correlative genes) [20]. DCA uses a zero-inflated negative binomial noise model to capture the nonlinear gene-gene dependencies to impute dropouts [20]. However, when the expression matrix is sparse, the expression levels of a gene in similar cells or the expression levels of similar genes in a cell are very likely to be affected by dropouts. In this case, these methods simply relying on similar cells or similar genes are incapable of acquiring sufficient information to infer the accurate imputed values.

To address this problem, several methods take into account both cell-level and gene-level information [21–23]. For instance, SAVER considers that gene expressions across cells obey the Poisson-gamma mixture distribution, and then borrows information across genes and cells by an empirical Bayes-like approach with a Poisson LASSO regression to impute dropouts [21]. SIMPLEs iteratively identifies correlated gene modules and cell clusters and imputes dropouts customized for individual gene module and cell type [22]. PBLR presents a cell sub-population based bounded low-rank method to impute the dropouts of scRNA-seq data, which uses the cell-level and gene-level information [23]. The ability to correctly identify dropouts is critical to the imputation methods. Besides the expression level of genes, the variation of gene expression is also important to describe the structural characteristics of dropouts. Moreover, a reasonable imputation method should take into account using the information unaffected by dropout events to implement imputation, which guarantees that no other noise is introduced in the imputation process.

We propose a statistical block imputation method SDImpute (S1 Fig). Firstly, SDImpute combines gene expression levels and the variations of gene expression across similar cells and similar genes to construct a dropout index matrix to identify dropout events and true zeros. Then, based on the Gaussian kernel coefficient matrix, SDImpute imputes dropouts by utilizing the weighted average of gene expression unaffected by dropouts from similar cells, which makes SDImpute recovering the data as well as maintaining the heterogeneity of gene expression across cells. The block imputation strategy of SDImpute reduces the program running time and memory cost. In the experiments, we compared SDImpute with the most widely used methods in both simulated datasets and real datasets, and the results show that SDImpute significantly improves the performance of the downstream analysis and outperforms the other imputation algorithms.

## Results

### Imputing dropouts and retaining true zeros

A reasonable imputation method should be capable of identifying the dropout events and recovering the dropout values without affecting the true zeros. As the bulk RNA-seq data results from the average gene expression of millions of cells and hardly suffers from dropouts, which is used to verify the ability of imputation methods in the matched imputed data [18–20].

**Fig 1:**
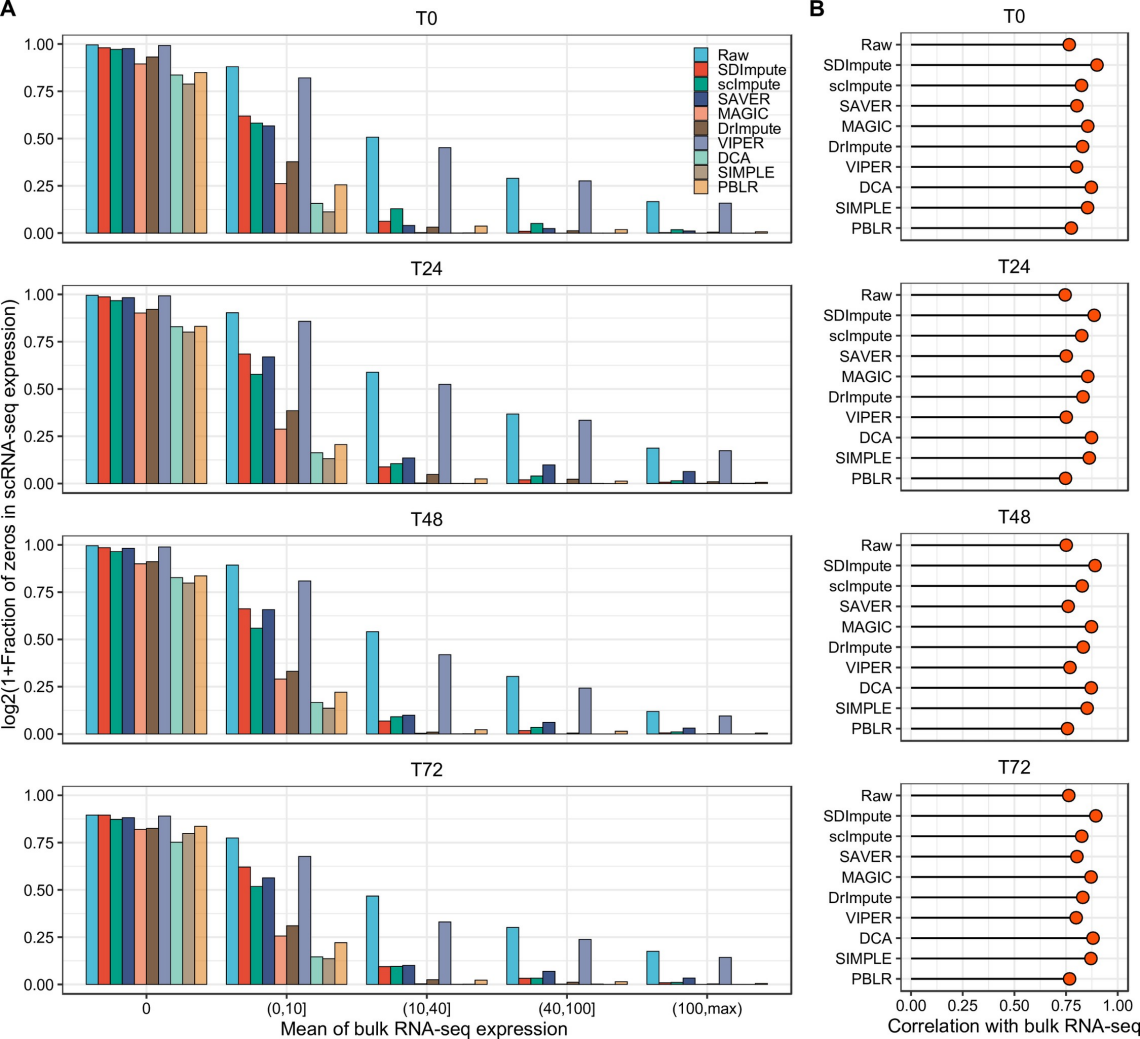
SDImpute imputes dropouts and retains true zeros in the Trapnell dataset. (A) The plots show the fraction of zero counts in scRNA-seq data against the mean of bulk expression entries across sample replicates of T0, T24, T36, and T72, respectively. The expression values are divided into five bins based on the mean of bulk gene expression entries of sample replicates. (B) Results of the Pearson Correlation between average expression levels of the same cell type in the imputed scRNA-seq data and the mean of the bulk RNA-seq dataset across sample replicates of T0, T24, T36, and T72, respectively.

We used the Trapnell dataset [24] containing both the scRNA-seq expression matrix and bulk RNA-seq expression matrix to demonstrate that SDImpute identified the dropouts and true zeros. Consistent results are presented in the plots at different times (Fig 1A). Against the mean of bulk expression entries across sample replicates, the raw expression matrix contains a large fraction of zeros, which likely corresponds to the dropout events. Here, we denoted the expression value ranging from 0 to 0.05 as a zero count, rendering minor flexibility to all imputation methods. Specifically, when the mean gene expression of the bulk data is zero (the first bins), the fractions of zero counts of the raw data are almost close to 1, which means these zero counts corresponding to true zero expressions. Interestingly, SDImpute, scImpute, SAVER, and VIPER also maintain the fractions of zeros close to 1 in the first bins, which means they successfully keep true zero counts unchanged. Moreover, the results of these methods show different drops of the fraction of zeros with the increase of the mean gene expression of the bulk data, yet VIPER maintains a high value on each bin and even matches the sizes of the raw data. Overall, SDImpute, scImpute, and SAVER are relatively conservative to impute the expression matrix. When the mean of bulk gene expression is greater than 10, SDImpute shows a more rapid decline than SAVER and scImpute, which means that SDImpute also performs a better imputation on the high expressed genes in the scRNA-seq data (Fig 1A). To make a further comparison, average expression levels of the same cell type in the imputed scRNA-seq data and the mean of the bulk RNA-seq dataset across sample replicates. Results show that all these methods improved the correlation levels, yet SDImpute and DCA provide better improvement than the other methods (Fig 1B).

### Improving the distribution and maintaining the heterogeneity of gene expression

To test the performance of SDImpute and other methods in maintaining gene expression heterogeneity, we utilized the Coefficient of Variance (CV) to measure the variation of gene expression within a cell subpopulation. Here, for a given gene in a cell subpopulation, we mainly analyzed the difference between the CV of expressions across cells after imputation and the CV of non-zero expressions before imputation in the following cases. Case 1: For a given gene, if the zero expressions within a cell subpopulation are all caused by dropouts, the CV of non-zero expressions in the raw data could explain the real variation of gene expression to a great extent. The imputed expressions of the gene would follow the same distribution as the non-zero expressions before imputation. In this case, the CV of expressions after imputation would be similar to the CV of non-zero expressions before imputation. Case 2: For a given gene, if the zero expressions within a cell subpopulation all correspond to the real zeros, the imputed data should remain these zeros unchanged. In this case, the distribution of non-zero expressions before imputation is different from that of all expressions after imputation. By computing, the CV of expressions after imputation would be higher than the CV of non-zero expressions before imputation (Proof is in S1 Text). Except for Case 1 and Case 2, for a given gene, if zero expressions within a cell subpopulation include both the dropouts and the real zeros, the imputation method should impute the dropout events and retain the real zero expressions. In this case, the CV of expressions after imputation would be also higher than the CV of non-zero expressions before imputation. In summary, for a given gene in a cell subpopulation, the CV of gene expressions across cells after imputation by a reasonable method would be either equal to or higher than the CV of non-zero expressions before imputation.

**Fig 2:**
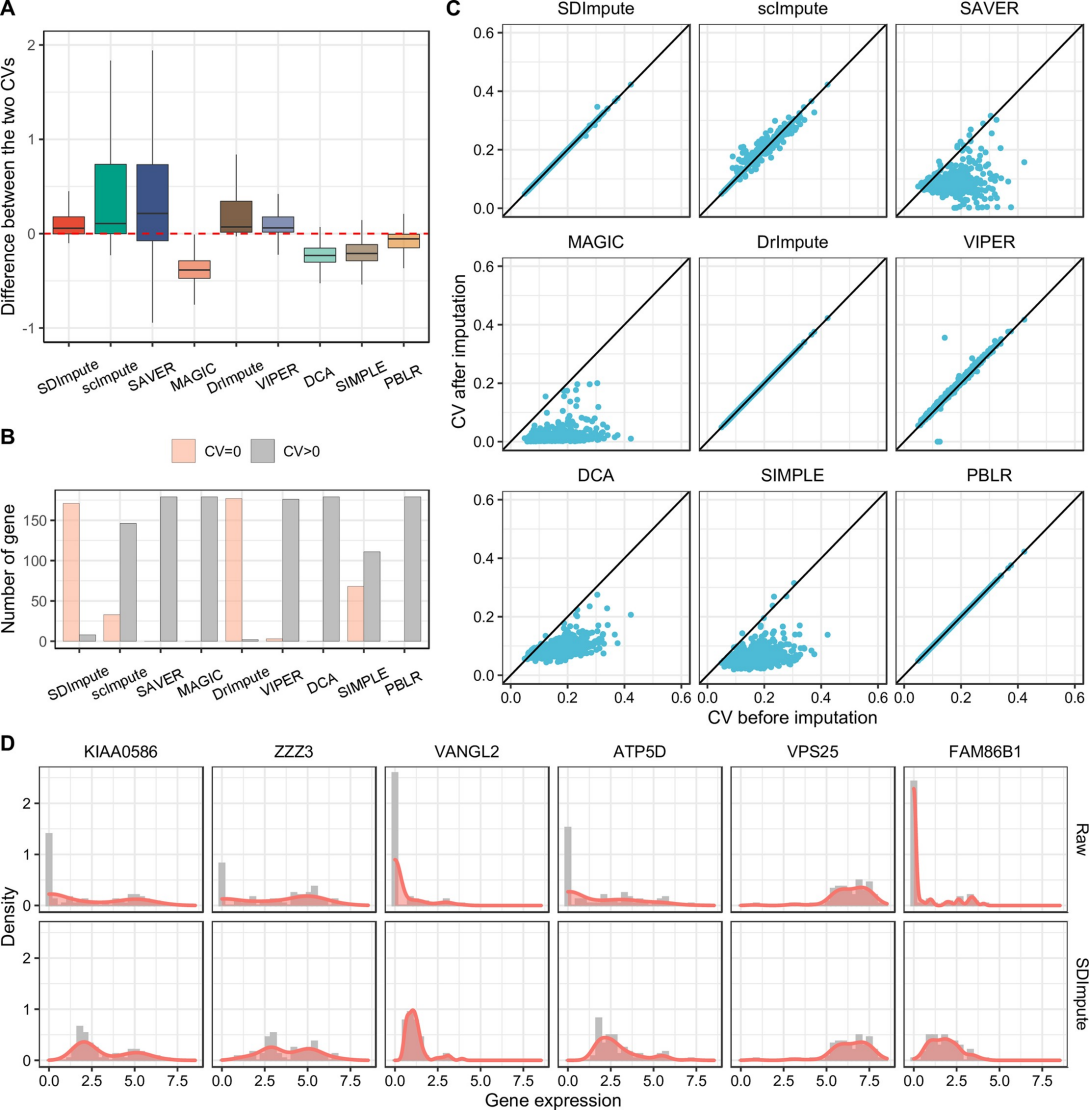
SDImpute improves the distribution and maintains the heterogeneity of gene expression in the Camp dataset. (A) Boxplots show the results of the difference between the CV of gene expressions after imputation and the CV of non-zero expressions (FPKM (fragment per kilobase million) is greater than 0) before imputation in DE cells. (B) The plot shows the results of the genes unexpressed across DE cells in the raw data. Here, the CV of unexpressed genes is defined as zero, and different colored bars show the number of these genes with the zero CV and non-zero CV in the imputed data, respectively. (C) Scatter plots show the results of the genes expressed in all DE cells before imputation. Here, the x-axis and y-axis represent the CV before imputation and the CV after imputation, respectively. (D) Density plots show the distribution of six genes across iPS cells in raw data vs imputed data by SDImpute.

We calculated the CV of non-zero expressions before imputation and the CV of all expressions after imputation in five cell subpopulations in the Camp dataset [25]. First of all, we used the box plot to show the distributions of the difference between the CV of non-zero expressions before imputation and the CV of all expressions after imputation. The results show that, for most genes, the difference values between the two CVs are non-negative in the imputed data by SDImpute, scImpute, SAVER, DrImpute, and VIPER (Figs 2A and S2–S5). However, for most genes, the CV after imputation is higher in the imputed data by either SAVER or scImpute, suggesting that SAVER or scImpute may treat most zeros as non-dropout events. Since the over-imputation may introduce artificial effects and influence downstream analyses, the imputation method should avoid this problem. For the genes unexpressed within a cell subpopulation in the raw data, we counted the number of the genes with non-zero CV and zero CV (CV of unexpressed genes is defined as zero) after imputation, respectively. Results show that SDImpute, DrImpute, and SIMPLE better keep these unexpressed genes within cell subpopulations (Figs 2B and S2–S5). On the other hand, for the genes that were all expressed in a cell subpopulation before imputation, they hardly suffered from dropouts. The imputation method should also avoid the over-imputation problem in this case. We used scatter plots to show the results of the two CVs for these genes within a cell subpopulation. Results show that SDImpute, VIPER, DrImpute, and PBLR keep the CV after imputation almost unchanged (Figs 2C and S2–S5). Moreover, the CV of gene expression also reflects the distribution of gene expression to a certain extent. To present the changes in the distribution of gene expression in the raw and imputed datasets, we randomly selected six genes to show their distributions across iPS cells in the Camp dataset. The results indicate that SDImpute and VIPER recover the great mass of dropout events and preserve the heterogeneity of gene expression across cells (Figs 2D and S6–S9). In particular, SDImpute, VIPER, and SIMPLE make the expression of VPS25 unaffected by dropouts unchanged (Figs 2D and S6–S9). Overall, SDImpute successfully maintains the heterogeneity of gene expression in single cells and avoids data over-imputation.

### Improving the separability and visualization of cell types

We used the visualization results of two simulated datasets and six datasets to show the capacity of SDImpute in the identification of cell types. Here, we colored each cell by its reference annotation.

**Fig 3:**
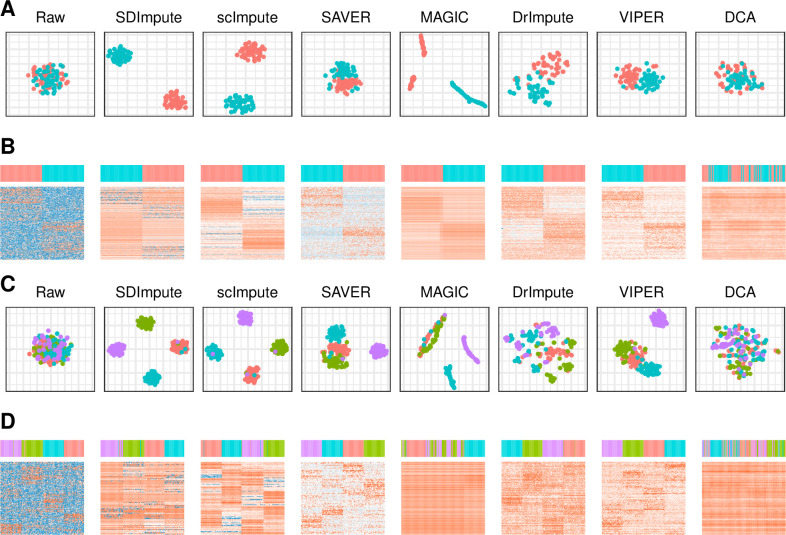
SDImpute improves the visualization of cell types in simulated datasets. (A), (C) Visualization after t-SNE [27] dimensionality reduction in simulated data of two cell types and four cell types, respectively. (B), (D) Heat maps of top 500 differential expression genes (DEGs) in simulated data of two cell types and four cell types, respectively.

We generated two simulated data by CIDR [26], one contains two cell types with 100 cells (8000 genes per cell), and the other one contains four cell types with 200 cells (8000 genes per cell). Specifically, SDImpute, scImpute, SIMPLE, and Drimpute achieve the separations of the different cell clusters in both two data (Fig 3A and 3C). Moreover, the heat maps show that the differences in gene expression of different cell types are highlighted by SDImpute, SIMPLE, and scImpute (Fig 3B and 3D).

**Fig 4:**
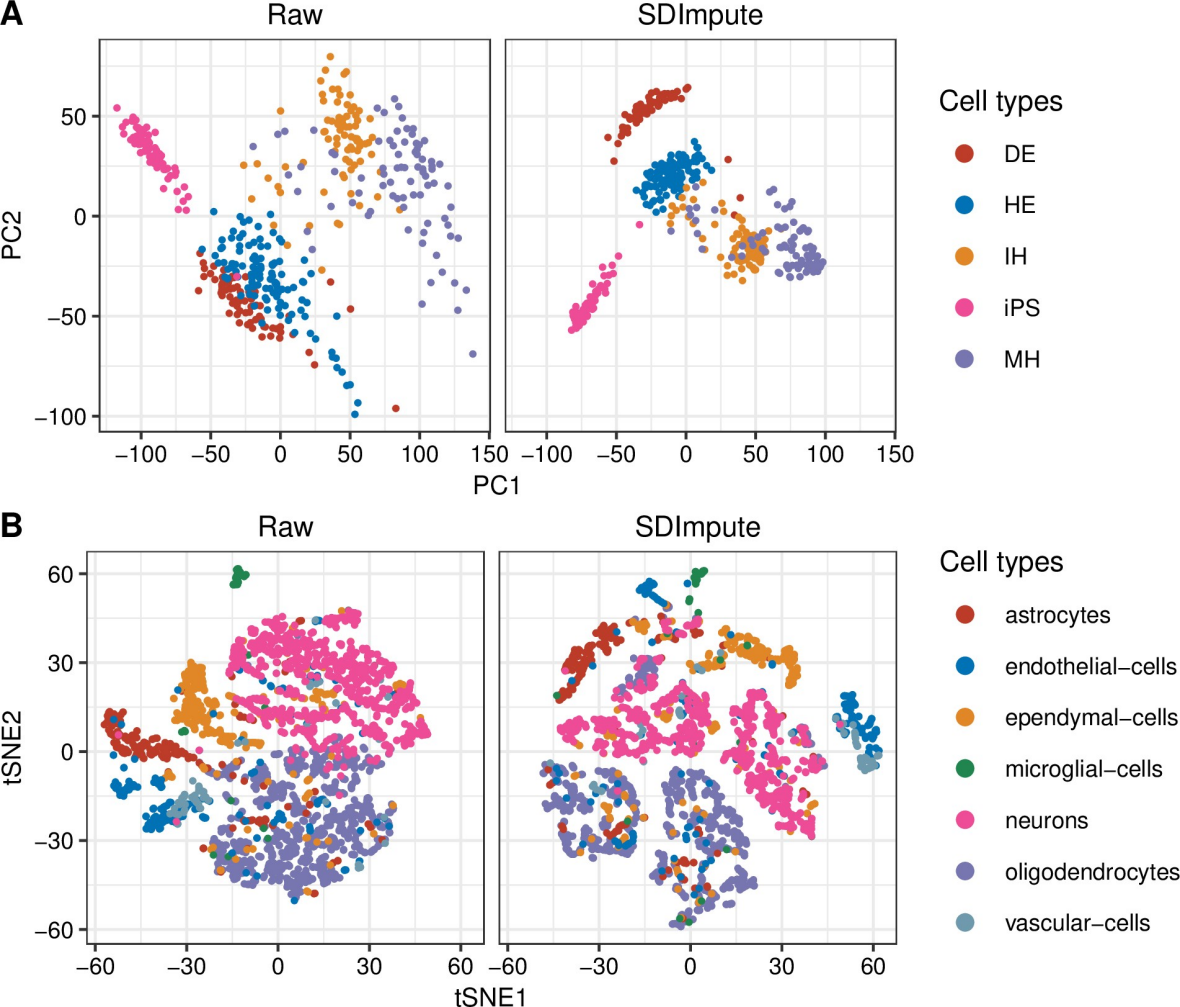
SDImpute improves the visualization of cell types in real datasets. (A) PCA plots in raw data and SDImpute imputed data of the Camp dataset. (B) t-SNE plots in raw data and SDImpute imputed data of the Romanov dataset.

We also checked the visualization results of datasets including the Camp dataset, Romanov dataset [28], Chu dataset (Cell Type and Time Course dataset) [29], Brain 9k dataset, and Trapnell dataset. Fig 4A shows the PCA plots of the first two PCs in the raw data and SDImpute imputed data of the Camp dataset. Since the raw data is affected by dropouts, cells are not well separated except for iPS cells. After SDImpute imputation, five cell clusters are separated from each other and more compact than in the raw data. Moreover, compared with the performance of other imputation methods in this dataset, only SDImpute Successfully separates DE cells from other cells (S10 Fig). SDImpute also improves the capacity of identifying cell types compared to the results in the raw data in the Romanov dataset. Specifically, SDImpute, PBLR, and scImpute make the astrocytes, oligodendrocytes, and neurons separate from other cell types (Figs 4B and S11). The same conclusions also are drawn in the Chu datasets, Brain 9k dataset, and Trapnell dataset, SDImpute improves the separability of different cell types (S12–S15 Figs).

### Improving the clustering accuracy of cells

To compare the clustering results, we used the Adjusted Rand Index (ARI), Jaccard Index, and Fowles Mallows (FM) Index to evaluate the relationship between the results of the k-means clustering algorithm and the reference labels of cells [30]. And the closer the indexes of ARI, Jaccard, and FM are to 1, the better the results of the clustering will be. In the k-means algorithm, the parameter K was set to the number of cell types of each dataset. As the k-means clustering algorithm is sensitive to the initial cluster centers selected randomly, we ran the clustering algorithm 1000 times and saved the results for analysis.

**Fig 5:**
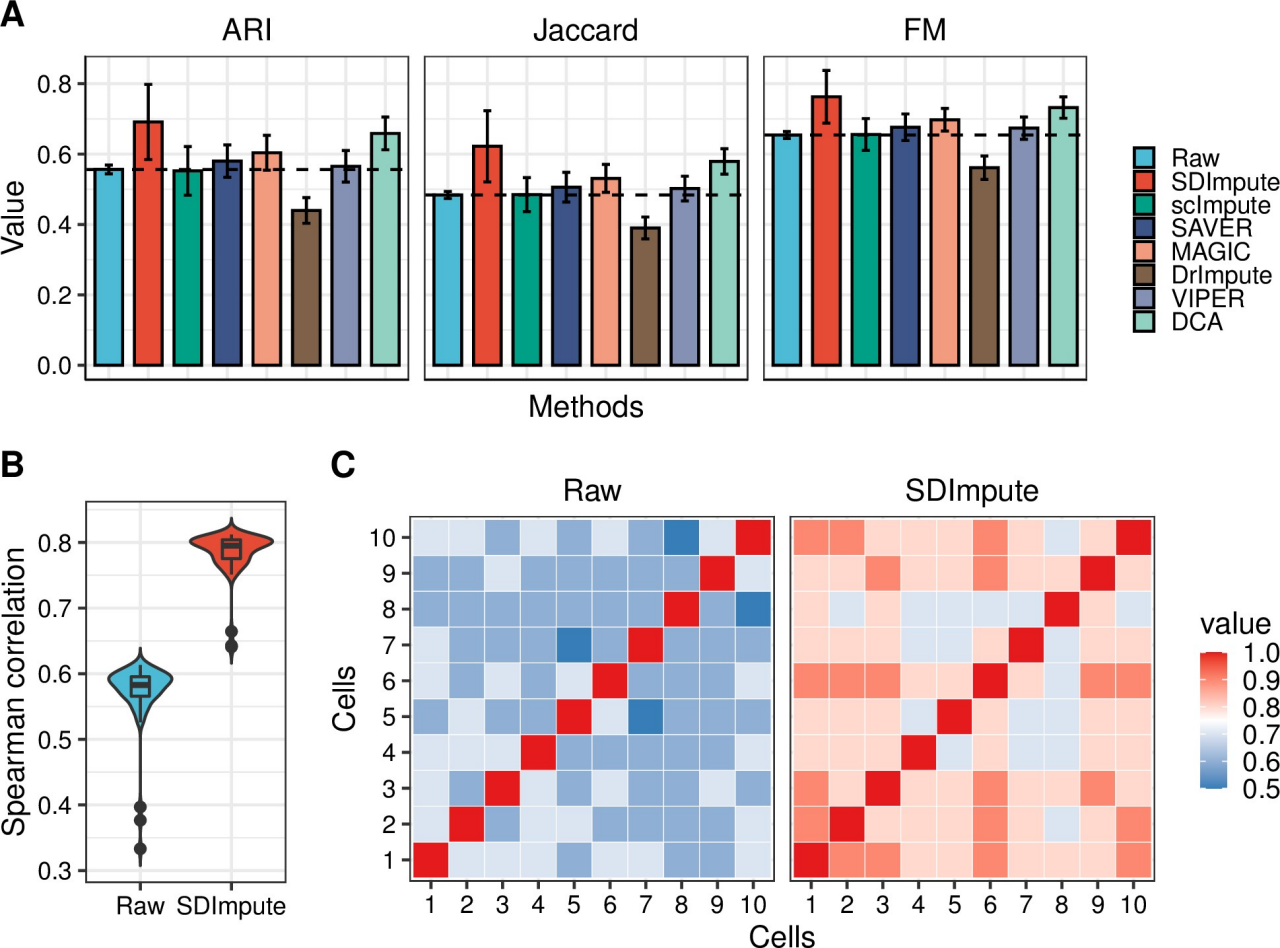
SDImpute improves the clustering accuracy in the Camp dataset. (A) Plots show the results of three clustering evaluation indexes, and the dashed line represents the clustering accuracy of raw data. (B) The plot shows the distribution of the Pearson correlation coefficient between definitive endoderm (DE) cells, and the Y-axis represents the mean of the correlation coefficients between each cell and the other cells. (C) Heat maps show the correlation coefficients between 10 randomly selected DE cells in the raw data and the SDImpute imputed data.

In order to get the cell clustering labels, we performed PCA and k-means clustering algorithm on the Camp dataset, Cell Type dataset, Time Course dataset, Romanov dataset, and Trapnell dataset. The results show that all these three indexes are improved by SDImpute, scImpute, SAVER, DrImpute, VIPER, DCA, and SIMPLE, yet SDImpute performs best among these methods in the Camp dataset (Fig 5A). After imputation, the improvements in the results of clustering are also shown in two simulated datasets (S16 and S17 Figs), the Romanov dataset (S18 Fig), the Chu dataset (Cell Type and Time Course dataset) (S19 and S20 Figs), Brain 9k dataset (S21 Fig), and the Trapnell dataset (S22 Fig). Moreover, we calculated the Pearson correlation coefficients between definitive endoderm (DE) cells in the Camp dataset, and the average correlation coefficient increased from 0.58 to 0.8 after imputation by SDImpute (Fig 5B). The heat map (Fig 5C) also shows the improvement of the correlations in the SDImpute imputed data, which is consistent with the results in Fig 5B.

### Improving the differential expression analysis of data

Differential expression analysis is an essential downstream analysis of the scRNA-seq data. Since the bulk RNA sequencing data is hardly affected by dropouts, the results of differential expression analysis in the imputed data should be consistent with those in the matched bulk RNA-seq data [19,20].

**Fig 6:**
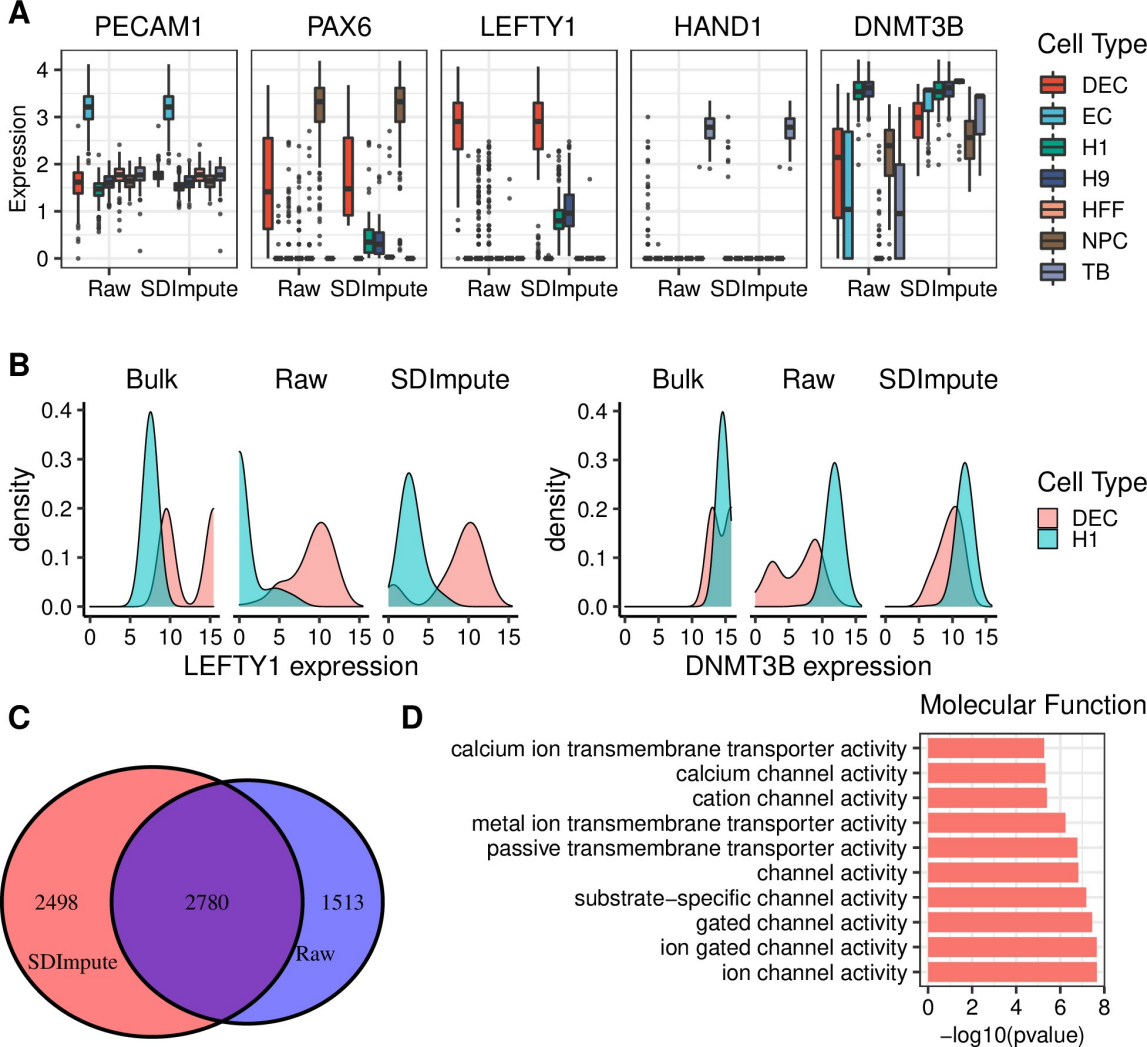
SDImpute improves differential expression analysis in the Cell Type dataset. (A) Box plots show expression levels of marker genes in raw data and SDImpute imputed data. (B) Density plots present the differential expression of two exemplary genes (LEFTY1 and DNMT3B) between H1 cells and DECs in the bulk data, raw data, and SDImpute imputed data, respectively. (C) Venn diagram of the differentially expressed genes (p-value <0.01) detected in raw data and SDImpute imputed data by DESeq2. (D) Enriched GO terms (p-value <10^−3^) related to the molecular function of the up-regulated genes of H1cells were only detected in SDImpute imputed data.

We used the Cell Type dataset containing scRNA-seq data and the bulk RNA-seq data to show the performance of SDImpute for differential expression analysis. As a result, the marker genes in the imputed data remain high expression levels in the corresponding cell cluster compared with the results of raw data, which implies that the imputed data do not affect the expression levels of the marker genes (Figs 6A and S23–S25). LEFTY1 is a marker gene of the endoderm derivatives cells (DEC) and a key gene in the development of the endoderm [29,31]. LEFTY1 should be highly expressed in non-differentiating H1 and H9 cells and turn off upon differentiation [32], and SDImpute does show a realistic recovery of expression that is biologically expected (Fig 6A). Moreover, the LEFTY1 expression level of bulk data is higher than that of the raw data in H1 cells, which implies that LEFTY1 expression in H1 cells is likely affected by dropouts in the scRNA-seq data (Figs 6B and S26). The expression level of LEFTY1 in H1 cells is increased after imputation by SDImpute, which makes it closer to the expression level in the bulk data. Similarly, DNMT3B is a marker gene of H1 cells [29], and its expression level in the SDImpute imputed data is closer to that in bulk data. Meanwhile, we used the R package DESeq2 [33] to identify differential expression genes (DEGs) between H1 cells and DECs. 2780 shared DEGs (p-value <0.01) genes are detected, and 2498 DEGs (p-value <0.01) genes only are identified by SDImpute imputed data (Fig 6C). Then, GO enrichment analysis was used to analyze up-regulated genes of H1 cells in the SDImpute imputed data, and some terms related to the function H1 cells were only detected in the SDImpute imputed data (Figs 6D and S27–S29). The results of the other imputation methods are presented in the S1–S10 Tables.

## Discussion

Since the scRNA-seq data suffers from dropout events that hinder the downstream analysis of data, we propose a statistical imputation method SDImpute to denoise the scRNA-seq data. SDImpute aims to implement data recovery and maintain the heterogeneity of gene expression across cells. One of the advantages of SDImpute practical application is that it is able to combine with the downstream analysis tools for the scRNA-seq data. In this paper, we performed downstream analysis experiments including clustering, visualization, and differential expression analysis in the simulated datasets and real datasets, and results showed that our method improved the results of the raw data and outperformed the other imputation methods. Moreover, in the results of the clustering and visualization analysis, SDImpute works well on both the UMI and non-UMI data and is robust to data size.

We also designed experiments to demonstrate that our imputation algorithm is robust to the parameters including *K* (the number of clusters), *T* (the dropout index candidate threshold), and *M* (the number of nearest neighbors). ARI, Jaccard Index, and FM Index were used to measure the clustering results of imputed data with different parameter values on the Camp dataset. For this dataset, the default values of parameter *K*, *T*, and *M* are 5, 0.5, and 10, respectively. In SDImpute, the value of parameter *K* is set either manually based on prior information of the input data or automatically obtained using the *kmeansruns* function in the *fpc* package (estimating parameter *K* by either average silhouette width or the Calinski Harabasz index). In the experiment, parameter *K* was taken from 3 to 12. Results show that all those parameter values improve the clustering accuracy except the smallest value 3 (S30 Fig). A reasonable explanation is that SDImpute imputes dropouts by borrowing information from similar cells based on the Gaussian kernel coefficient matrix. That is, the nearer cells will get larger weight coefficients, and they play an important role in the imputation process for the missing values. As long as the candidate set of nearest similar cells for each cell is stable, the result will be relatively stable. The parameter *T* mainly controls the degree of imputation to the gene expression matrix. We randomly select eight cells and eight genes from the Camp data to present the distributions of dropout index, and the dropout index of each expression is very close to either zero or one (S31 Fig). Moreover, the clustering evaluation indexes of 9 different parameter *T* values (0.1 to 0.9) are much the same except extreme values (0.1 and 0.9) at both ends (S32 Fig). The results show that SDImpute is relatively robust to the selection of parameter *T*, and the recommended value of parameter *T* is 0.5. Moreover, the results of parameter *M* show that 8 different values (5 to 40) improve the clustering accuracy to almost the same degree (S33 Fig). When the number of nearest neighbors for each cell is small, the parameter *M* should not be too large to guarantee that it makes sense. In general, it is recommended to set this parameter to an integer between 10 and 30.

In the future, for the scRNA-seq data which isolated and captured cells from continuous processes such as organization differentiation trajectories, we will consider the expression of single cells in a one-dimensional manifold based on SDImpute [10,34,35]. In other words, we will take into account the information on the time dimension in the imputation process.

## Materials and methods

### Datasets

Six scRNA-seq datasets and two simulated datasets were utilized to evaluate and compare the performance of different imputation methods. The scRNA-seq data measured by two types of experimental platforms, including Fluidigm platform (non-UMI based protocols) and 10X Genomics platform (UMI based protocols). And a summary of the scRNA-seq datasets is shown in Table 1.

**Table 1:** A summary of the scRNA-seq datasets.

Datasets	Cells	Cell types	Cell source	Date type
Trapnell [24]	362	4	Human myoblasts	non-UMI
Camp [25]	425	5	Human liver bud cells	non-UMI
Romanov [28]	2,881	7	Mus musculus brain cells	non-UMI
Chu (Cell Type) [29]	1,018	7	Human embryonic stem cells	non-UMI
Chu (Time Course) [29]	758	6	Human definitive endoderm cells	non-UMI
Brain 9k	9,128	13	E18 mouse brain cells	UMI

The details of six scRNA-seq datasets are as follows. i) Trapnell et al. provide a scRNA-seq dataset for primary human myoblasts, the dataset contains both scRNA-seq and bulk RNA-seq expression matrices, and sequenced cells were captured over a time-course of serum-induced differentiation [24]. The dataset is available at Gene Expression Omnibus with the accession number GSE52529. ii) The Camp dataset contains single-cell transcriptome from pluripotent to hepatocyte-like lineages at multiple points in time in two-dimensional culture [25]. The dataset is available at Gene Expression Omnibus with the accession number GSE81252. iii) Romanov et al. sampled single cells randomly from a central column of the medial-ventral diencephalon and sorted 2881 cells into seven major cell types [28]. The dataset is available at Gene Expression Omnibus with the accession number GSE74672. iv) Chu et al. sequenced a total of 1018 human embryonic stem cells and 758 time-course profiled single cells and provided matched population bulk RNA-seq samples for both the human embryonic stem cells and time-course profiling [29]. The dataset is available at Gene Expression Omnibus with the accession number GSE75748. v) Brain 9k dataset provided UMI-based scRNA-seq for E18 mouse brain cells obtained from hippocampus, cortex, and subventricular zone. The dataset is available from the 10X Genomics webpage (https://www.10xgenomics.com/).

The simulated scRNA-seq datasets were generated by using the *scSimulator* function of R package cidr (version 0.1.5). The first dataset of two cell types consists of 100 cells and 8000 genes, each cell type contains 50 cells. The parameters were set as follows: *N = 2*, *nDG = 500*, *nMK = 10*, *nNDG = 7480*, *k = 50*, *logmean = 5*.*25*, *logsd = 1*, *v = 9*.*2*. Another dataset of four cell types consists of 200 cells and 10000 genes, each type contains 50 cells. The parameters were set as follows: *N = 4*, *nDG = 500*, *nMK = 10*, *nNDG = 9460*, *k = 50*, *logmean = 5*.*25*, *logsd = 1*, *v = 9*.*2*.

### Data preprocessing

The input data of SDImpute is a *I*×*J* gene expression matrix, columns and rows represent cells and genes respectively. Firstly, the raw count matrix *X*^*C*^ is normalized, the result matrix denoted as *X*^*N*^:

$$X_{ij}^{N}=\frac{X_{ij}^{C}\cdot 10^{6}}{\sum_{k=1}^{J}X_{ik}^{C}},i=1,2,\cdots ,I,j=1,2,\cdots ,J,$$

where *i* represents the i-th gene and *j* represents the j-th cell. Then the matrix *X* is obtained by logarithmic transformation of the normalized matrix *X*^*N*^:

$$X_{ij}=\log_{2}(X_{ij}^{N}+1),i=1,2,\cdots ,I,j=1,2,\cdots ,J,$$

where the constant 1 is added to avoid infinite values during the transformation.

### Identification of dropouts and true zeros

To find similar cells between cells roughly, SDImpute firstly applies Principal Component Analysis (PCA) on the matrix *X*, then utilizes the clustering algorithm k-means on the result matrix of PCA to cluster the cells into *K* groups. We denote *C*_*j*_ = *k* if cell *j* belongs to the cell cluster *k*(*k* = 1,2,⋯,*K*), and define the candidate similar cell set of cell *j* as

$$S_{j}=\{j^{\prime }|C_{j^{\prime }}=C_{j},j^{\prime }\neq j\}.$$

Meanwhile, according to the clustering results of cells, the gene expression matrix *X* is divided into *K* blocks, denoted as *X*^(1)^,*X*^(2)^,⋯,*X*^(*K*)^, where *X*^(*k*)^(*k* = 1,2,⋯,*K*) is the k-th block with *I* by *J*_*k*_ dimensions, and *J*_1_+*J*_2_+⋯+*J*_*K*_ = *J*. SDImpute identifies dropouts in each block respectively.

Instead of considering all zero or low expression values as dropout events, SDImpute combines the information of cell-level and gene-level to determine whether a zero expression represents a dropout. SDImpute mainly uses the expression level and local variation to model the dropout index for each gene. First of all, in each block, the average gene expression levels and the ratios of zero count are fitted to a decreasing logistic regression function by non-linear Least Square Method [36]. This model assumes an empirical relationship between mean expression values and dropout rates. Thus the estimation of empirical dropout rate $EP_{ij}^{\left(k\right)}$ for $X_{ij}^{\left(k\right)}$ (the expression of gene *i* in cell *j* which belongs to block *k*) is obtained. Nevertheless, using the model based on the expression levels alone hardly distinguish the dropout events well from the true zeros, a more informative and accurate identification method for dropout events is necessary. As the dropout event occurs when gene expression is observed at a medium or even high expression level in most cells but is not detected in a few cells [36]. That is, when a gene has high expression value and low variation in most cells, a zero count is more likely to present a dropout event. Conversely, when a gene has continuous low expression and high variation across cells, a zero count may reflect the real biological variability [19,36]. Therefore the variation of gene expression in both cellular and genetic dimensions is also taken into account to describe the structural characteristics of dropout events. Here, the variation of gene expression in each block is presented by the coefficient of variation (CV) of genes, which is a normalized measure of the dispersion degree of a probability distribution. It is a dimensionless measure and is defined as the ratio of the standard deviation to mean value:

$$CV_{i}^{\left(k\right)}=\frac{D(X_{i,}^{\left(k\right)})}{E(X_{i,}^{\left(k\right)})+\theta },$$

$$E(X_{i,}^{\left(k\right)})=\frac{1}{J_{k}}\sum_{j=1}^{J_{k}}X_{ij}^{\left(k\right)},$$

$$D(X_{i,}^{\left(k\right)})=\sqrt{\frac{1}{J_{k}}\sum_{j=1}^{J_{k}}(X_{ij}^{\left(k\right)}-E(X_{i,}^{\left(k\right)}){)}^{2}},$$

where $CV_{i}^{\left(k\right)}$ denotes the coefficient of variation of gene *i* in the block *k*, and $D(X_{i,}^{\left(k\right)})$ and $E(X_{i,}^{\left(k\right)})$ denote the standard deviation and the mean of the expression for gene *i* across all cells from the block *k* respectively, and *θ* is a constant to make sense in the denominator. Then, the $CV_{i}^{\left(k\right)}$ value is normalized to a value between 0 and 1 by the inverse tangent function, denoted as ${\tilde{CV}}_{i}^{\left(k\right)}$:

$${\tilde{CV}}_{i}^{\left(k\right)}=\frac{2}{\pi }\arctan(CV_{i}^{\left(k\right)}\cdot \lambda^{\left(k\right)}),$$

$$\lambda^{\left(k\right)}=\sqrt{\frac{1}{I}\sum_{i=1}^{I}(CV_{i}^{\left(k\right)}-\frac{1}{I}\sum_{i=1}^{I}CV_{i}^{\left(k\right)}{)}^{2}},$$

where *λ*^(*k*)^ represents the standard deviation of the coefficient of variation of all the genes in the k-th block. Combining the empirical dropout rate with the coefficient of variation of gene expression, we get a dropout index for each gene expression $X_{ij}^{\left(k\right)}$, denoted as

$$DI_{ij}^{\left(k\right)}=EP_{ij}^{\left(k\right)}\cdot {\tilde{CV}}_{i}^{\left(k\right)}.$$

Thus the gene expression matrix *X* corresponds to a dropout index matrix *DI* with the same dimension.

Let *T* be the dropout index candidate threshold. If *DI*_*ij*_≤*T*, no imputation is require for *X*_*ij*_; if *DI*_*ij*_>*T*, the expression needs to be imputed. Meanwhile, for gene *i*, the candidate similar cell set of cell *j* which is unaffected by dropout events is obtained, and denoted as

$$N_{i-j}=\{j^{\prime }|j^{\prime }\in S_{j},DI_{ij^{\prime }}\leq T\}.$$

### Block imputation for the dropout events

Based on the result matrix of PCA, the cell distance matrix *D* is calculated. To reasonably assign weights to similar cells, the Gaussian kernel function is used to calculate the coefficient matrix. Because the Gaussian kernel function is a nonlinear decreasing function of distance, it means that the closer cells will get larger weights and the farther cells will get smaller weights. The Gaussian kernel coefficient matrix *G* is obtained based on the matrix *D*, the component of *G* is

$$G_{mn}=\exp(-(\frac{D_{mn}}{\sigma_{m}}{)}^{2}),$$

$$\sigma_{m}=E(D_{m,S_{m}^{*}}),$$

where *D*_*mn*_ represents the Euclidean distance between cell *m* and cell *n*, and *m* = 1,2,⋯,*J*,*n* = 1,2,⋯,*J*, the kernel width value *σ*_*m*_ is set as the mean of the distances to the nearest neighbors of the cell *m*, $S_{m}^{*}$ represents the set of *M* nearest neighbors to the cell *m*. Instead of fixing a single value, *G* adapts kernel width value for each cell based on the local density of cells. The kernel is narrow in dense areas and wide in sparse areas, which reduces the effect of imbalance in the density of cells.

For the gene expression which is influenced by dropout event, namely the corresponding dropout index satisfies *DI*_*ij*_>*T*, SDImpute imputes them and leaves other values unchanged. The corresponding block of Gaussian kernel coefficient matrix is taken as the weight matrix. Then SDImpute uses the weight average of the gene expression unaffected by dropouts as imputation value for dropout event. The imputed gene expression matrix $\hat{X}$ is

$${\hat{X}}_{ij}=\left\{ \begin{array}{l} X_{ij},\;DI_{ij}<T,\\ W(G_{i,N_{i-j}},X_{i,N_{i-j}}),\;DI_{ij}\geq T. \end{array}\right.$$

Where *W* is the weighted average function, $G_{i,N_{i-j}}$ and $X_{i,N_{i-j}}$ are the Gaussian kernel coefficient vector and gene expression vector of gene *i* across all cells of set *N*_*i*−*j*_ respectively.

## Supporting information

S1 Text The supplemental proof. (PDF)

S1 Fig The workflow figure of SDimpute. (TIF)

S2 Fig SDImpute improves the distribution and maintains the heterogeneity of gene expression across HE cells in the Camp dataset. (TIF)

S3 Fig SDImpute improves the distribution and maintains the heterogeneity of gene expression across IH cells in the Camp dataset. (TIF)

S4 Fig SDImpute improves the distribution and maintains the heterogeneity of gene expression across iPS cells in the Camp dataset. (TIF)

S5 Fig SDImpute improves the distribution and maintains the heterogeneity of gene expression across MH cells in the Camp dataset. (TIF)

S6 Fig The distribution of six genes (choose at random) across iPS cells in raw data vs imputed data by DrImpute, VIPER, and DCA. (TIF)

S7 Fig The distribution of six genes (choose at random) across iPS cells in raw data vs MAGIC imputed data. (TIF)

S8 Fig The distribution of six genes (choose at random) across iPS cells in raw data vs imputed data by SAVER and scImpute. (TIF)

S9 Fig The distribution of six genes (choose at random) across iPS cells in raw data vs imputed data by SAVER and scImpute. (TIF)

S10 Fig The PCA results calculated on imputed datasets by various methods in the Camp dataset. (TIF)

S11 Fig The t-SNE results calculated on imputed data by various methods in the Romanov dataset. (TIF)

S12 Fig The t-SNE results calculated on imputed data by various methods in the Time Course dataset. (TIF)

S13 Fig The t-SNE results calculated on imputed data by various methods in the Cell Type dataset. (TIF)

S14 Fig The t-SNE results calculated on imputed data by various methods in the Brain 9K dataset. (TIF)

S15 Fig The t-SNE results calculated on imputed data by various methods in the Trapnell dataset. (TIF)

S16 Fig The results of clustering evaluation indexes (ARI, Jaccard, and FM) in the simulated dataset (two cell types). (TIF)

S17 Fig The results of clustering evaluation indexes (ARI, Jaccard, and FM) in the simulated dataset (four cell types). (TIF)

S18 Fig The results of clustering evaluation indexes (ARI, Jaccard, and FM) in the Romanov datasets. (TIF)

S19 Fig The results of clustering evaluation indexes (ARI, Jaccard, and FM) in the Time Course datasets. (TIF)

S20 Fig The results of clustering evaluation indexes (ARI, Jaccard, and FM) in the Cell Type datasets. (TIF)

S21 Fig The results of clustering evaluation indexes (ARI, Jaccard, and FM) in the Brain 9K dataset. (TIF)

S22 Fig The results of clustering evaluation indexes (ARI, Jaccard, and FM) in the Trapnell dataset. (TIF)

S23 Fig The expression levels of some marker genes in raw data comparing with imputed data by scImpute, SAVER, DrImpute. (TIF)

S24 Fig The expression levels of some marker genes in raw data comparing with imputed data by MAGIC, VIPER, and DCA. (TIF)

S25 Fig The expression levels of some marker genes in raw data comparing with imputed data by SIMPLE, and PBLR. (TIF)

S26 Fig Two exemplary genes LEFTY1 and DNMT3B in the bulk data, raw data, and imputed data by various methods. (TIF)

S27 Fig Enriched GO terms (p < 10^−3^) are only detected in the up-regulated genes of H1 cells in the SDimpute imputed data. (TIF)

S28 Fig Enriched GO terms (p < 10^−3^) are detected in the up-regulated genes of H1 cells in the raw data. (TIF)

S29 Fig Enriched GO terms (p < 10^−3^) are detected in the up-regulated genes of H1 cells in SDimpute imputed data. (TIF)

S30 Fig The sensitivity analysis of parameter K. (TIF)

S31 Fig The distribution of dropout index in the Camp dataset. (TIF)

S32 Fig The sensitivity analysis of parameter T. (TIF)

S33 Fig The sensitivity analysis of parameter M. (TIF)

S1 Table Enriched GO terms (p < 10^−3^) detected in the up-regulated genes of H1 cells in raw data. (XLSX)

S2 Table Enriched GO terms (p < 10^−3^) detected in the up-regulated genes of H1 cells in SDImpute imputed data. (XLSX)

S3 Table Enriched GO terms (p < 10^−3^) detected in the up-regulated genes of H1 cells in scImpute imputed data. (XLSX)

S4 Table Enriched GO terms (p < 10^−3^) detected in the up-regulated genes of H1 cells in SAVER imputed data. (XLSX)

S5 Table Enriched GO terms (p < 10^−3^) detected in the up-regulated genes of H1 cells in MAGIC imputed data. (XLSX)

S6 Table Enriched GO terms (p < 10^−3^) detected in the up-regulated genes of H1 cells in DrImpute imputed data. (XLSX)

S7 Table Enriched GO terms (p < 10^−3^) detected in the up-regulated genes of H1 cells in VIPER imputed data. (XLSX)

S8 Table Enriched GO terms (p < 10^−3^) detected in the up-regulated genes of H1 cells in DCA imputed data. (XLSX)

S9 Table Enriched GO terms (p < 10^−3^) detected in the up-regulated genes of H1 cells in SIMPLE imputed data. (XLSX)

S10 Table Enriched GO terms (p < 10^−3^) detected in the up-regulated genes of H1 cells in PBLR imputed data. (XLSX)
