# Correction: SDImpute: A statistical block imputation method based on cell-level and gene-level information for dropouts in single-cell RNA-seq data

**DOI:** 10.1371/journal.pcbi.1009770

**Published:** 2022-01-05

**Authors:** Jing Qi, Yang Zhou, Zicen Zhao, Shuilin Jin

There is an error in Figs 3 and 5. The correct figures can be seen below:

**Fig 3:**
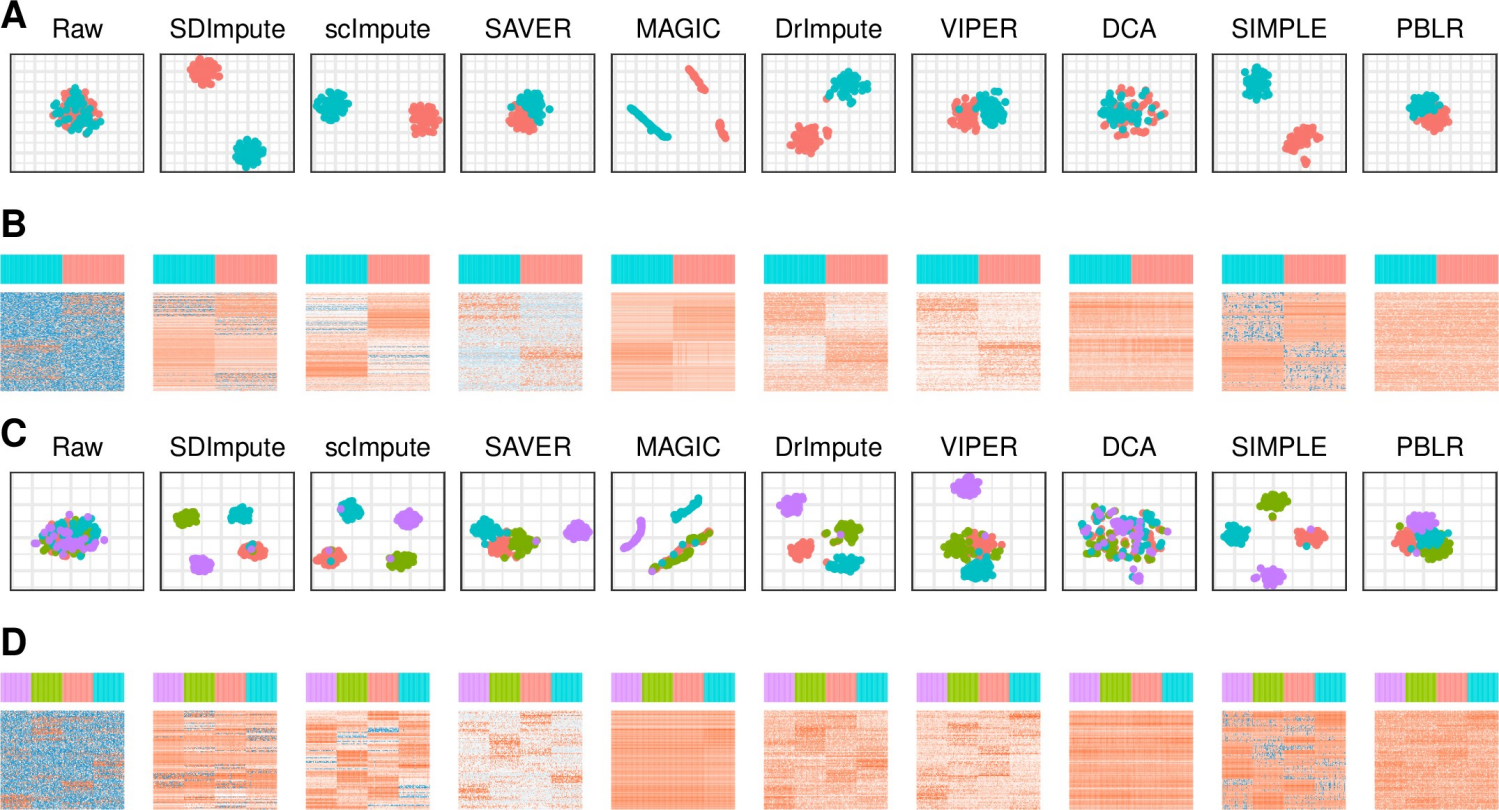
SDImpute improves the visualization of cell types in simulated datasets. (A), (C) Visualization after t-SNE [27] dimensionality reduction in simulated data of two cell types and four cell types, respectively. (B), (D) Heat maps of top 500 differential expression genes (DEGs) in simulated data of two cell types and four cell types, respectively.

**Fig 5:**
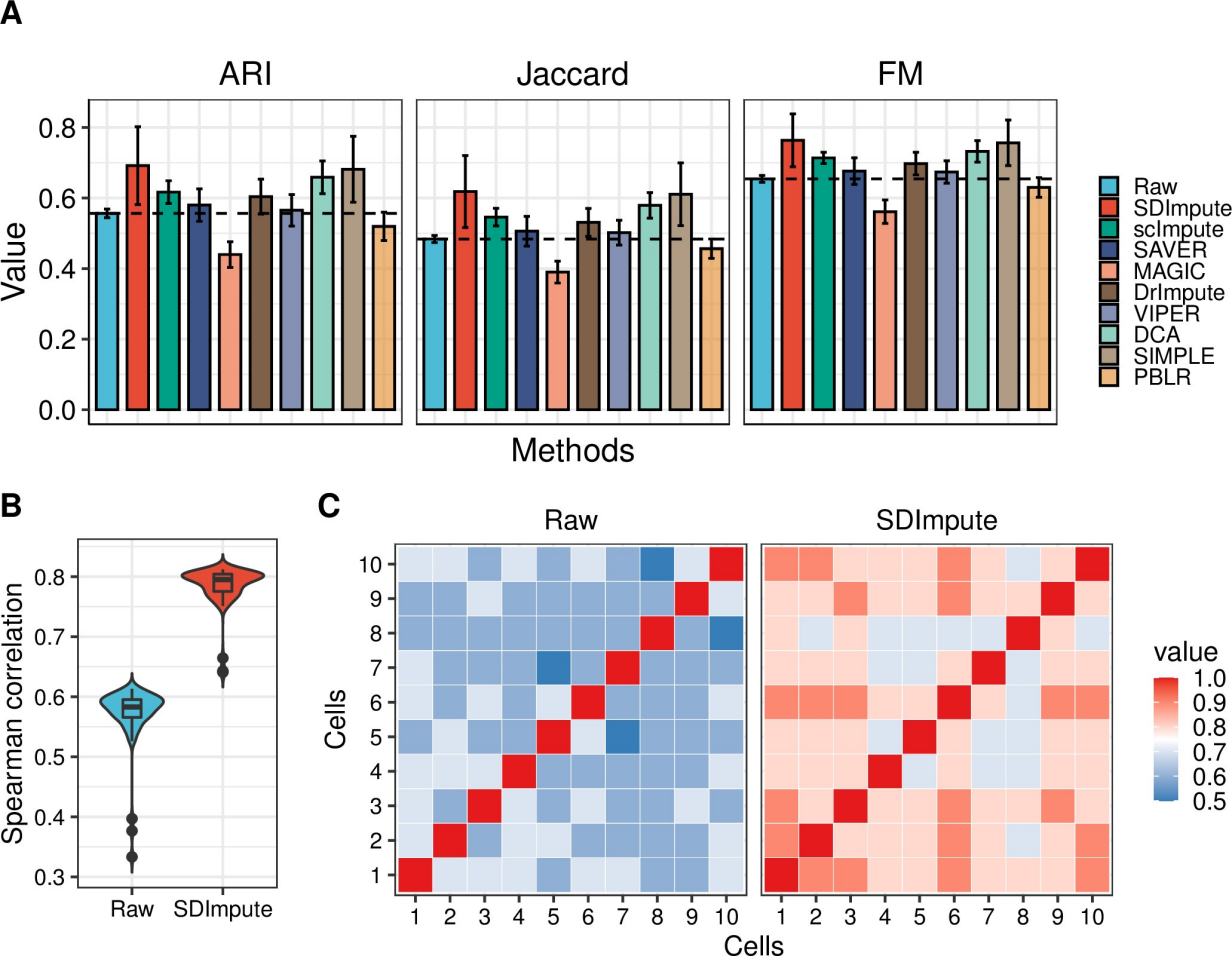
SDImpute improves the clustering accuracy in the Camp dataset. (A) Plots show the results of three clustering evaluation indexes, and the dashed line represents the clustering accuracy of raw data. (B) The plot shows the distribution of the Pearson correlation coefficient between definitive endoderm (DE) cells, and the Y-axis represents the mean of the correlation coefficients between each cell and the other cells. (C) Heat maps show the correlation coefficients between 10 randomly selected DE cells in the raw data and the SDImpute imputed data.
