# Supplementary material for: SDImpute: A statistical block imputation method based on cell-level and gene-level information for dropouts in single-cell RNA-seq data
Source: PLoS Comput Biol. 2021 Jun 17;17(6):e1009118. doi: 10.1371/journal.pcbi.1009118 (PMC8266063; doi:10.1371/journal.pcbi.1009118)
Supplement: S1 Text — (PDF) [file pcbi.1009118.s001.pdf]

Supporting Text

S1 Text. The supplemental proof.

Proposition 1: The coefficient of variation of $n(n \in N^*, n \geq 2)$ real positive variables is smaller than the coefficient of variation of the n positive variables and $m(m \in N^*)$ zeros.

Proof: The $n(n \in N^*, n \geq 2)$ positive variables are denoted as $x_1, x_2, \cdots, x_n$, and the CV of the n positive variables is:

$$CV_{(n)} = \frac{\sqrt{\frac{1}{n-1}\sum_{i=1}^{n}(x_i - \frac{1}{n}\sum_{i=1}^{n} x_i)^2}}{\frac{1}{n}\sum_{i=1}^{n} x_i}$$
$$= \sqrt{\frac{n^2}{n-1} \cdot \frac{\sum_{i=1}^{n} x_i^2}{(\sum_{i=1}^{n} x_i)^2} - \frac{n}{n-1}}.$$

The n positive variables and m zeros are denoted as $x_1, x_2, \cdots, x_n, x_{n+1}, \cdots, x_{n+m}$, where $x_{n+1} = x_{n+2} = \cdots = x_{n+m} = 0$, and the CV of the $m+n$ variables is:

$$CV_{(m+n)} = \frac{\sqrt{\frac{1}{n+m-1}\sum_{i=1}^{n+m}(x_i - \frac{1}{n+m}\sum_{i=1}^{n+m} x_i)^2}}{\frac{1}{n+m}\sum_{i=1}^{n+m} x_i}$$
$$= \sqrt{\frac{(n+m)^2}{n+m-1} \cdot \frac{\sum_{i=1}^{n} x_i^2}{(\sum_{i=1}^{n} x_i)^2} - \frac{n+m}{n+m-1}}.$$

We want to prove that $CV_{(m+n)} > CV_{(n)}$.

Firstly, let $\frac{\sum_{i=1}^{n} x_i^2}{(\sum_{i=1}^{n} x_i)^2} = A > 0$, $p(y) = \frac{y^2}{y-1}A$, $q(y) = -\frac{y}{y-1}$, and $f(y) = p(y) + q(y)$, then $CV_{(n)} = \sqrt{f(n)}$.

It is obvious that $q(y)$ is an increasing function when $y \geq 2$. Then, calculate the first derivative of $p(y)$:

$$p'(y) = A(1 - \frac{1}{(y-1)^2}) = A\frac{y(y-2)}{(y-1)^2}.$$

When $y \geq 2$, $p'(y) \geq 0$, thus $p(y)$ is an increasing function if $y \geq 2$.

As $m+n > n$, thus $\sqrt{f(m+n)} > \sqrt{f(n)}$, and $CV_{(m+n)} > CV_{(n)}$.
