# Supplementary figures and images for: SDImpute: A statistical block imputation method based on cell-level and gene-level information for dropouts in single-cell RNA-seq data
Source: PLoS Comput Biol. 2021 Jun 17;17(6):e1009118. doi: 10.1371/journal.pcbi.1009118 (PMC8266063; doi:10.1371/journal.pcbi.1009118)

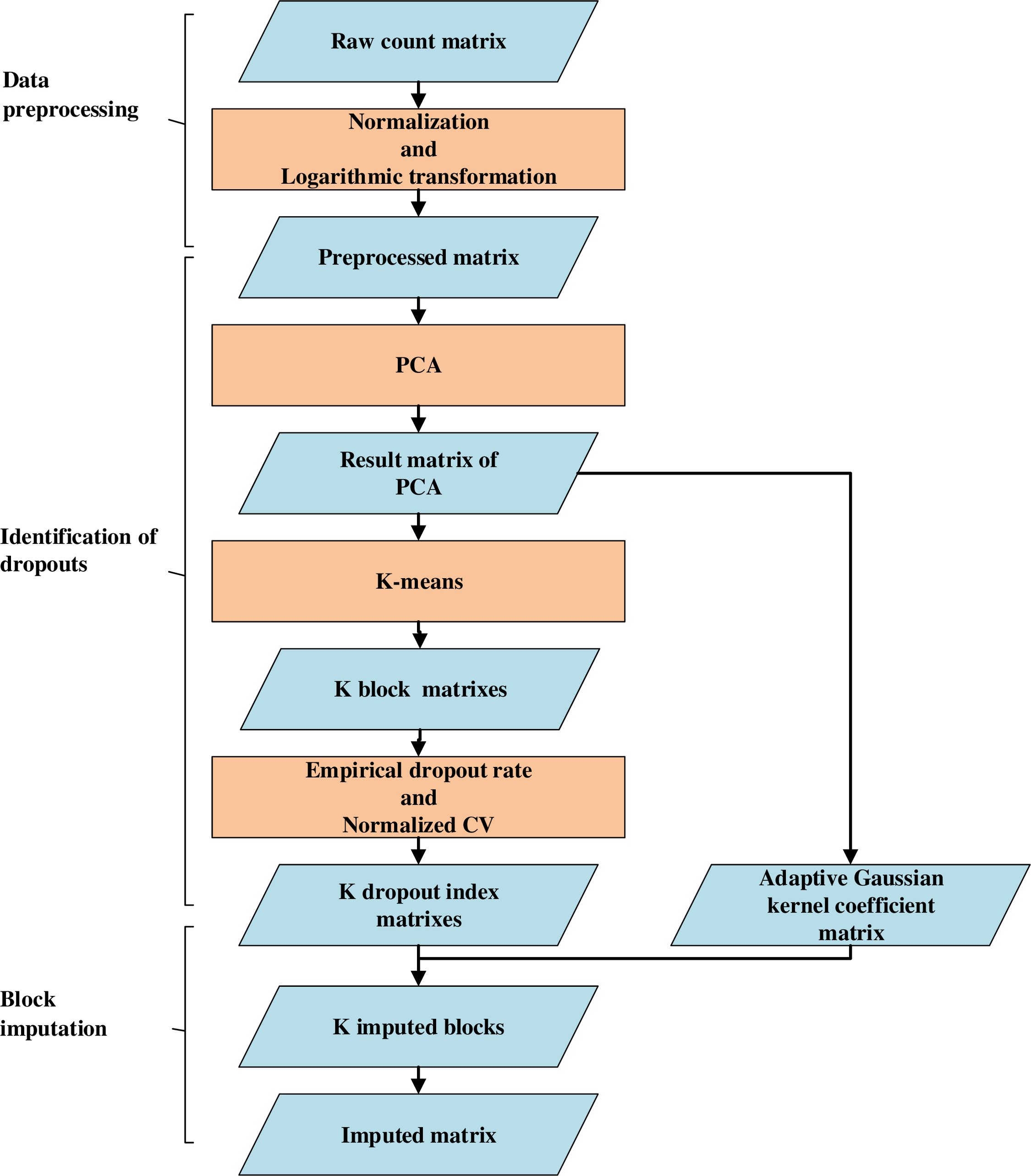

Supplement: S1 Fig — (TIF) [file pcbi.1009118.s002.tif]

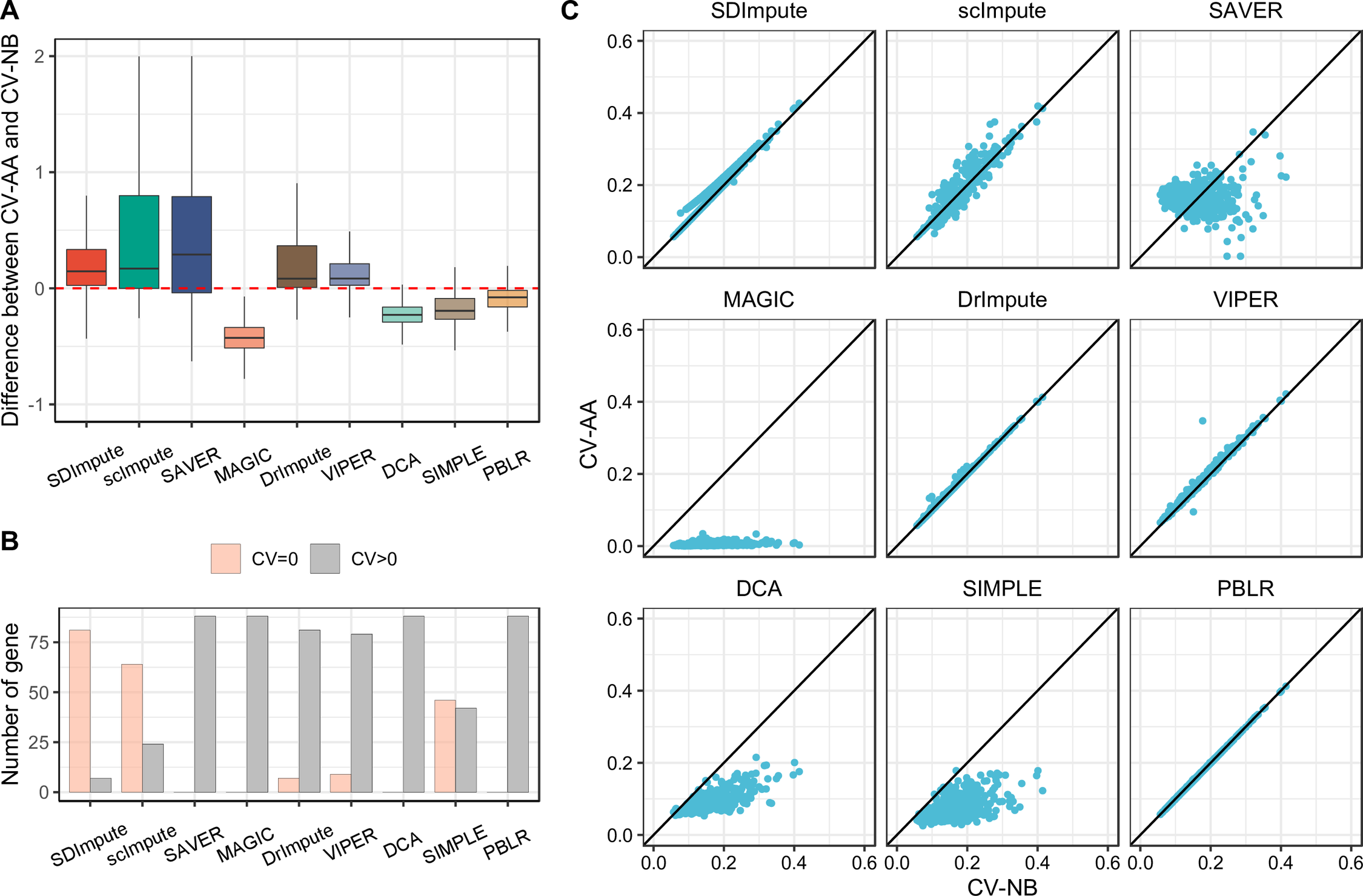

Supplement: S2 Fig — (TIF) [file pcbi.1009118.s003.tif]

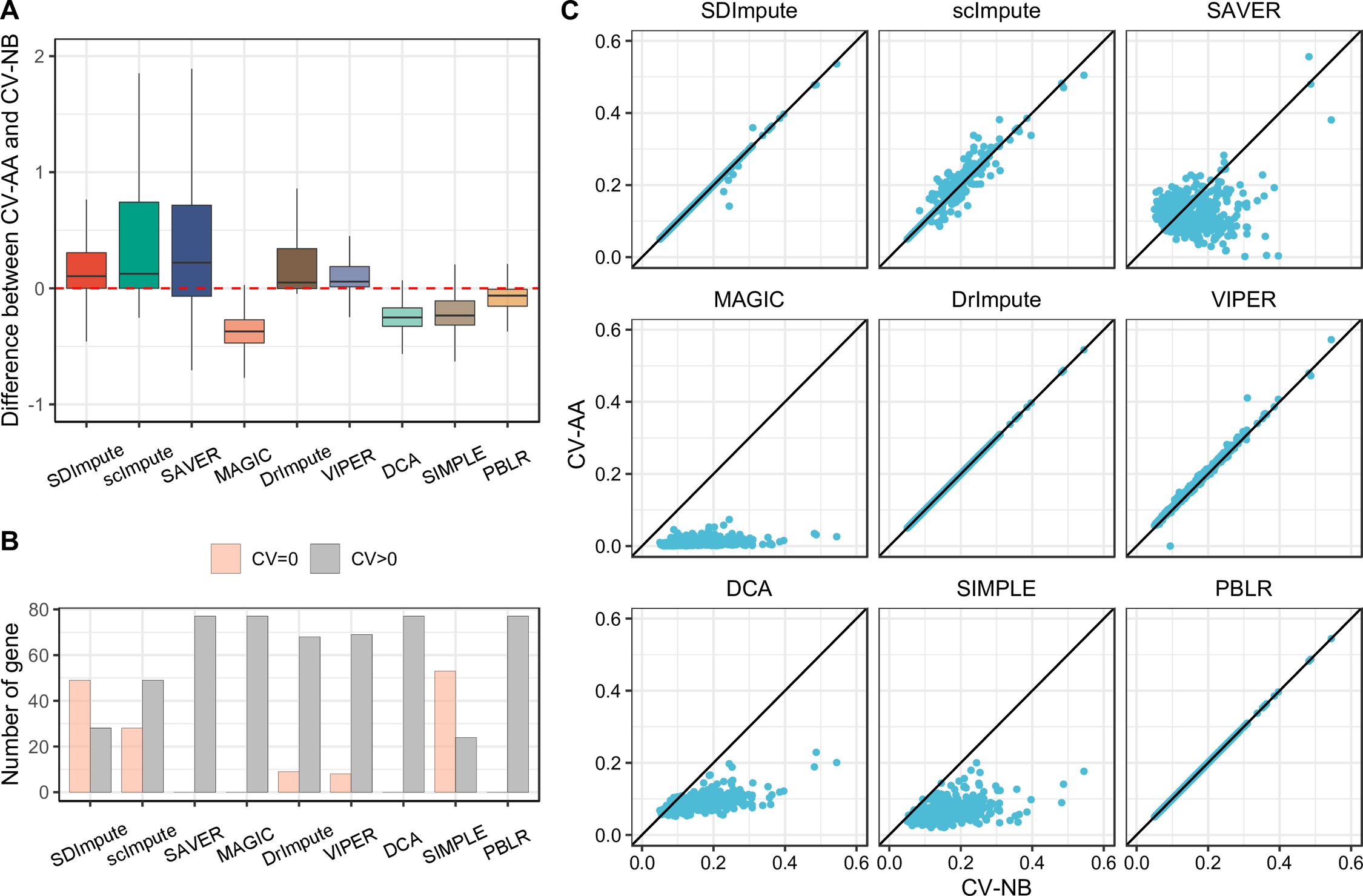

Supplement: S3 Fig — (TIF) [file pcbi.1009118.s004.tif]

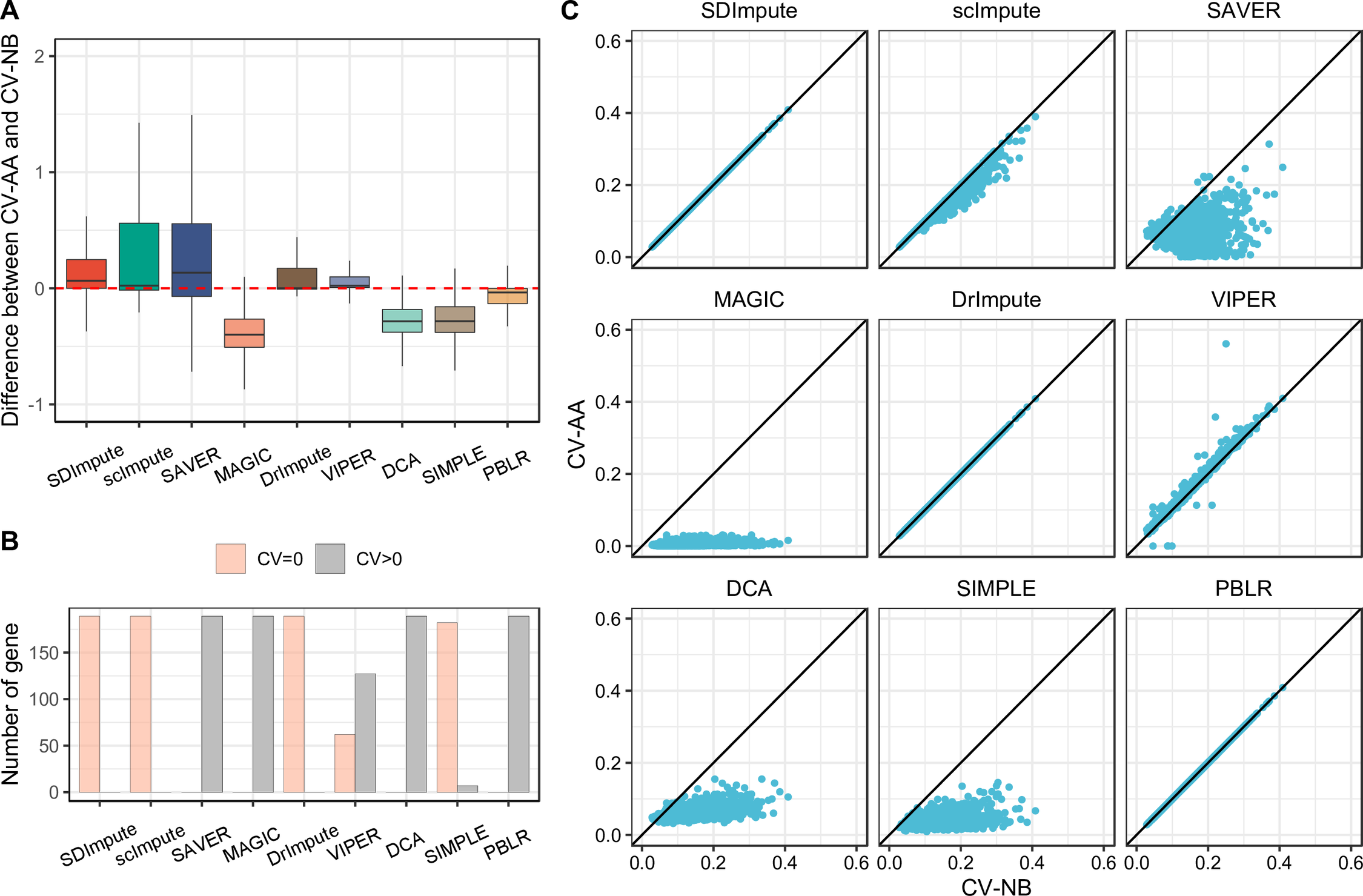

Supplement: S4 Fig — (TIF) [file pcbi.1009118.s005.tif]

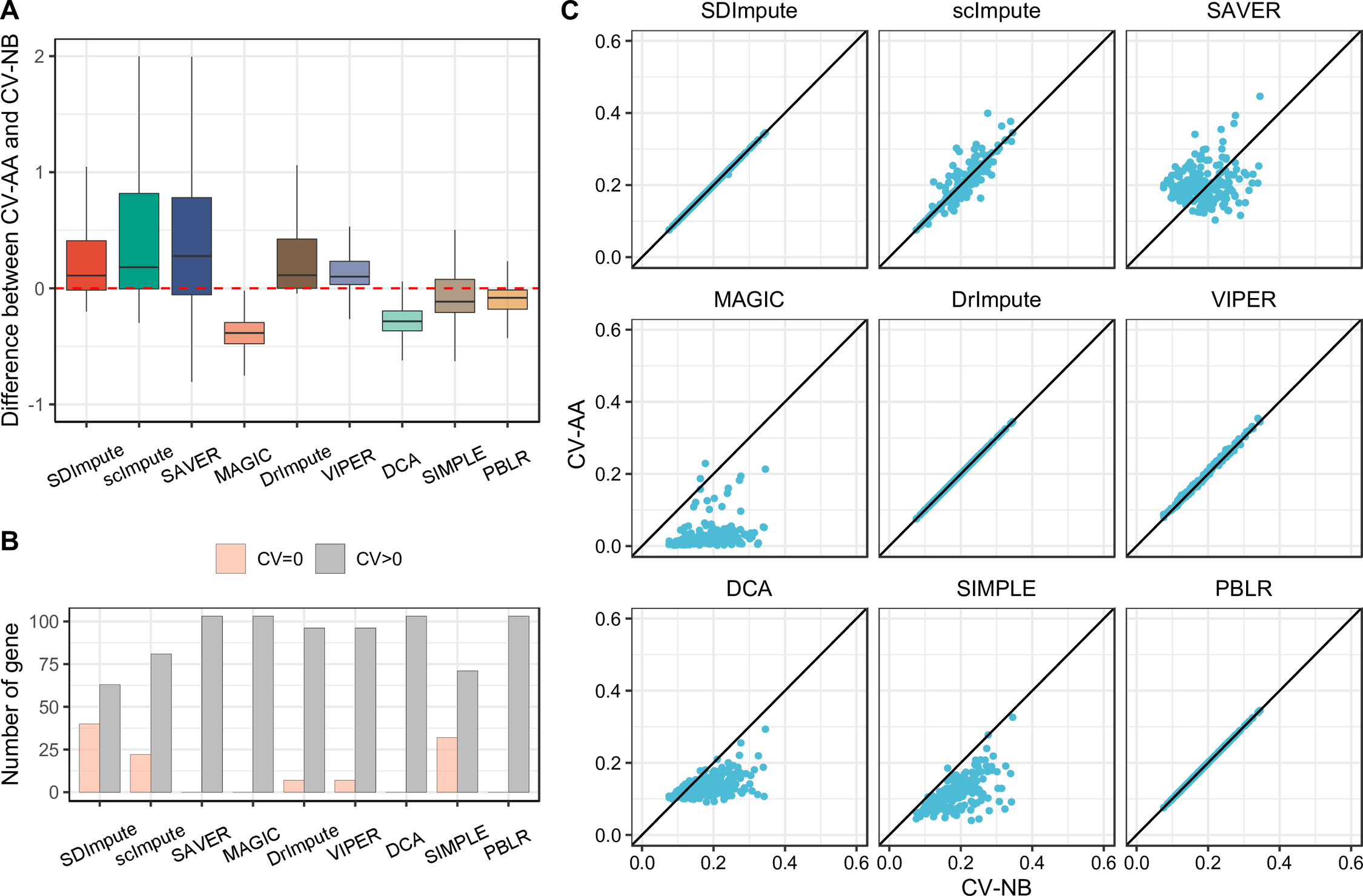

Supplement: S5 Fig — (TIF) [file pcbi.1009118.s006.tif]

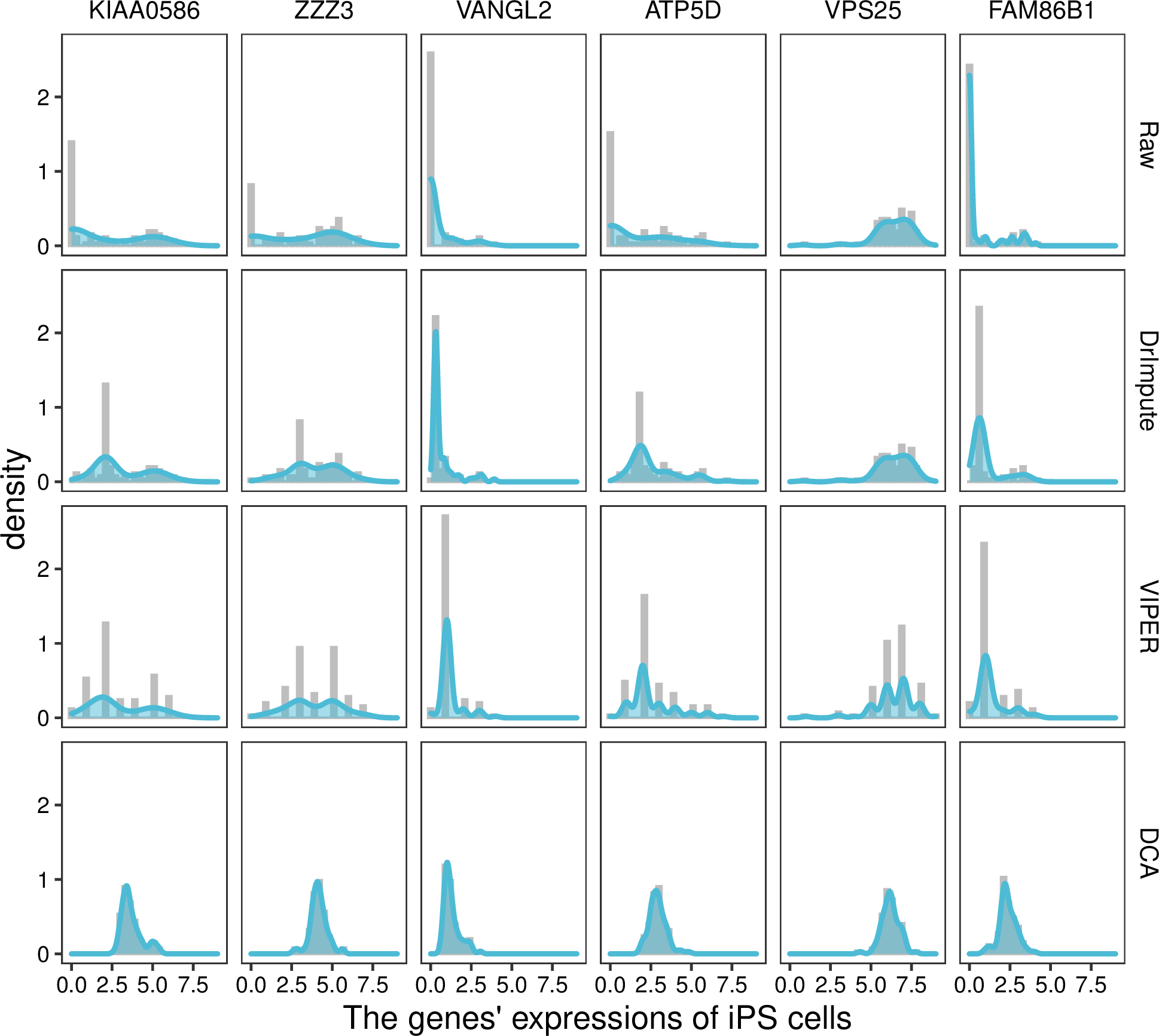

Supplement: S6 Fig — (TIF) [file pcbi.1009118.s007.tif]

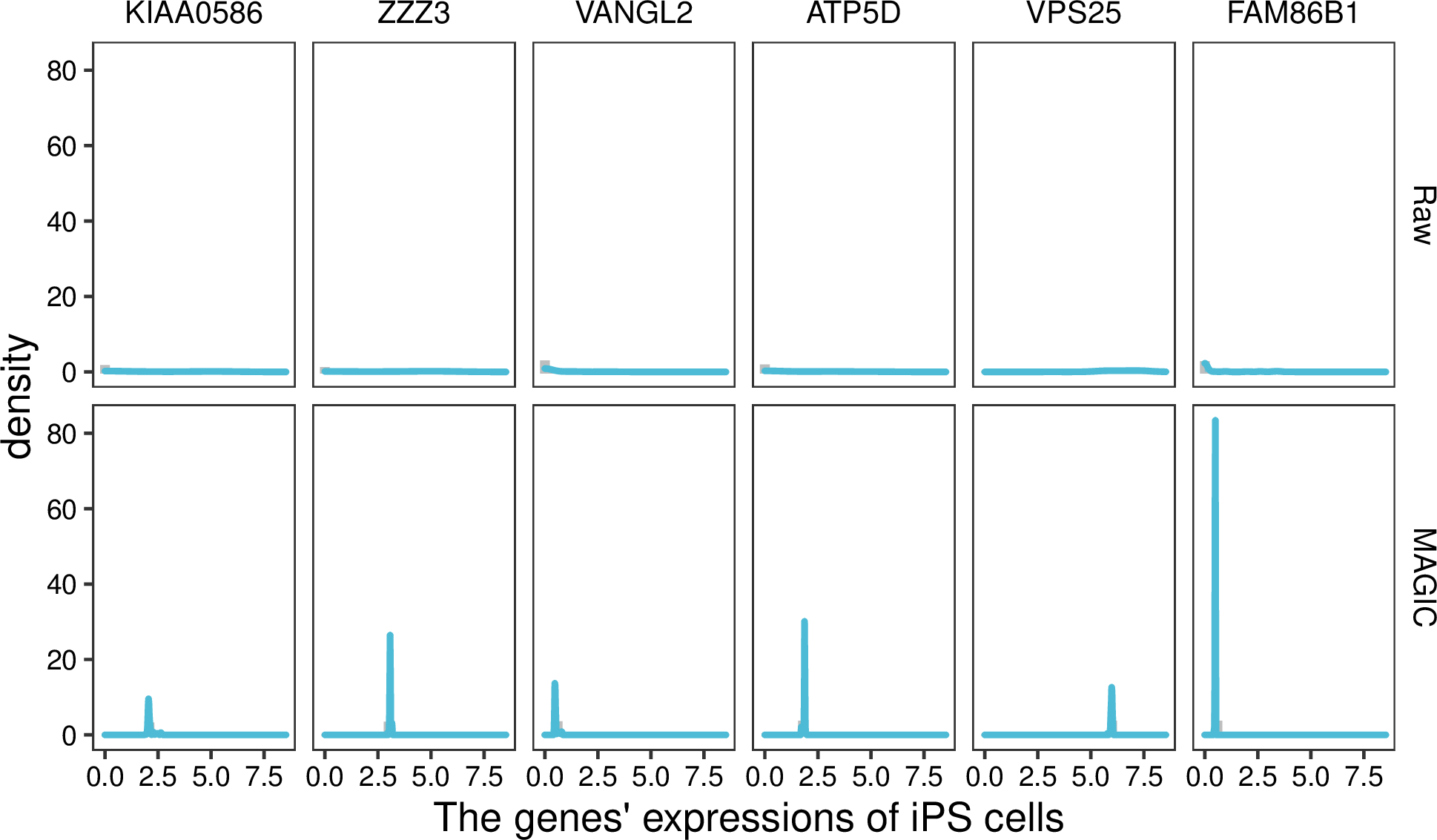

Supplement: S7 Fig — (TIF) [file pcbi.1009118.s008.tif]

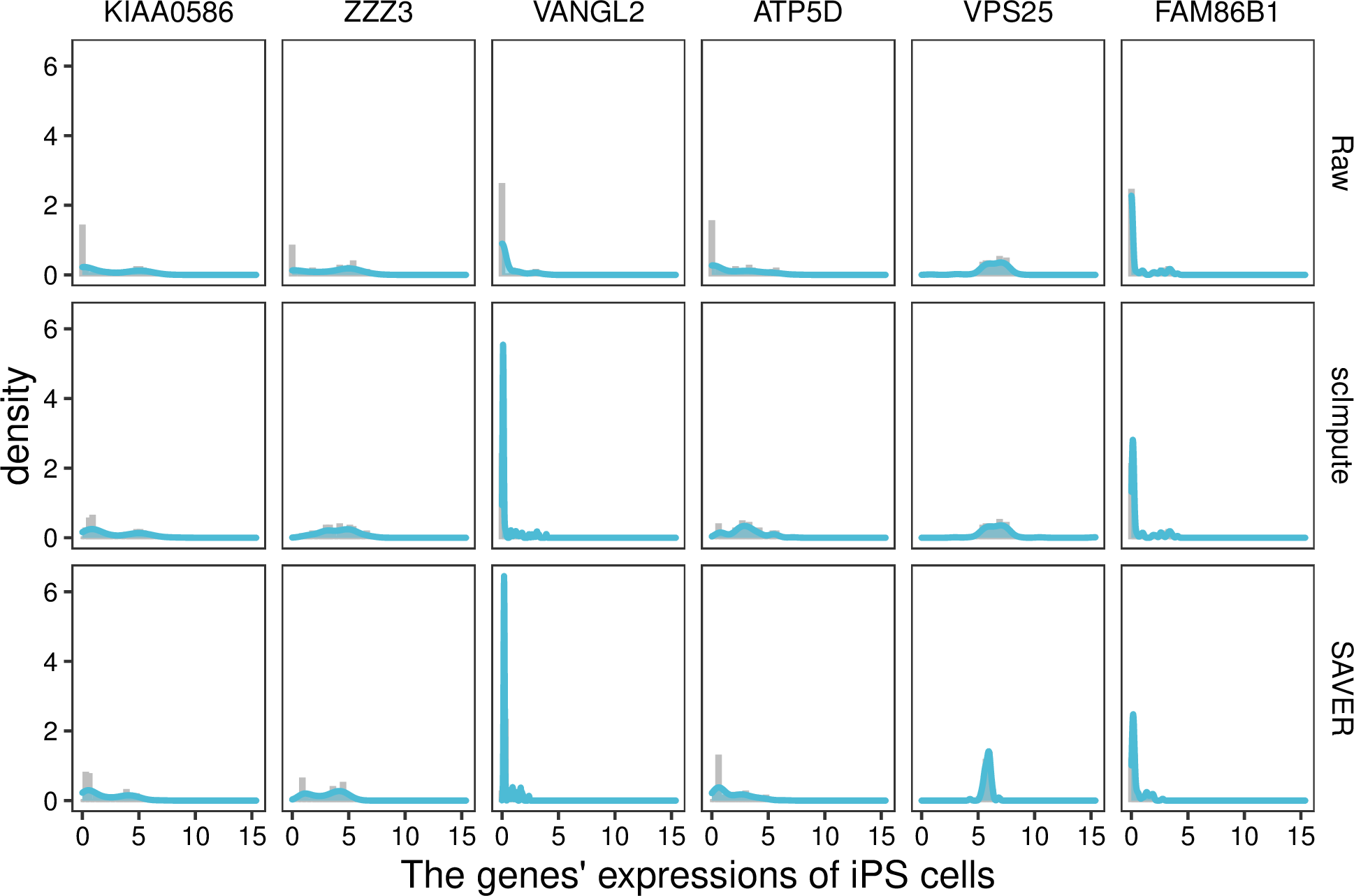

Supplement: S8 Fig — (TIF) [file pcbi.1009118.s009.tif]

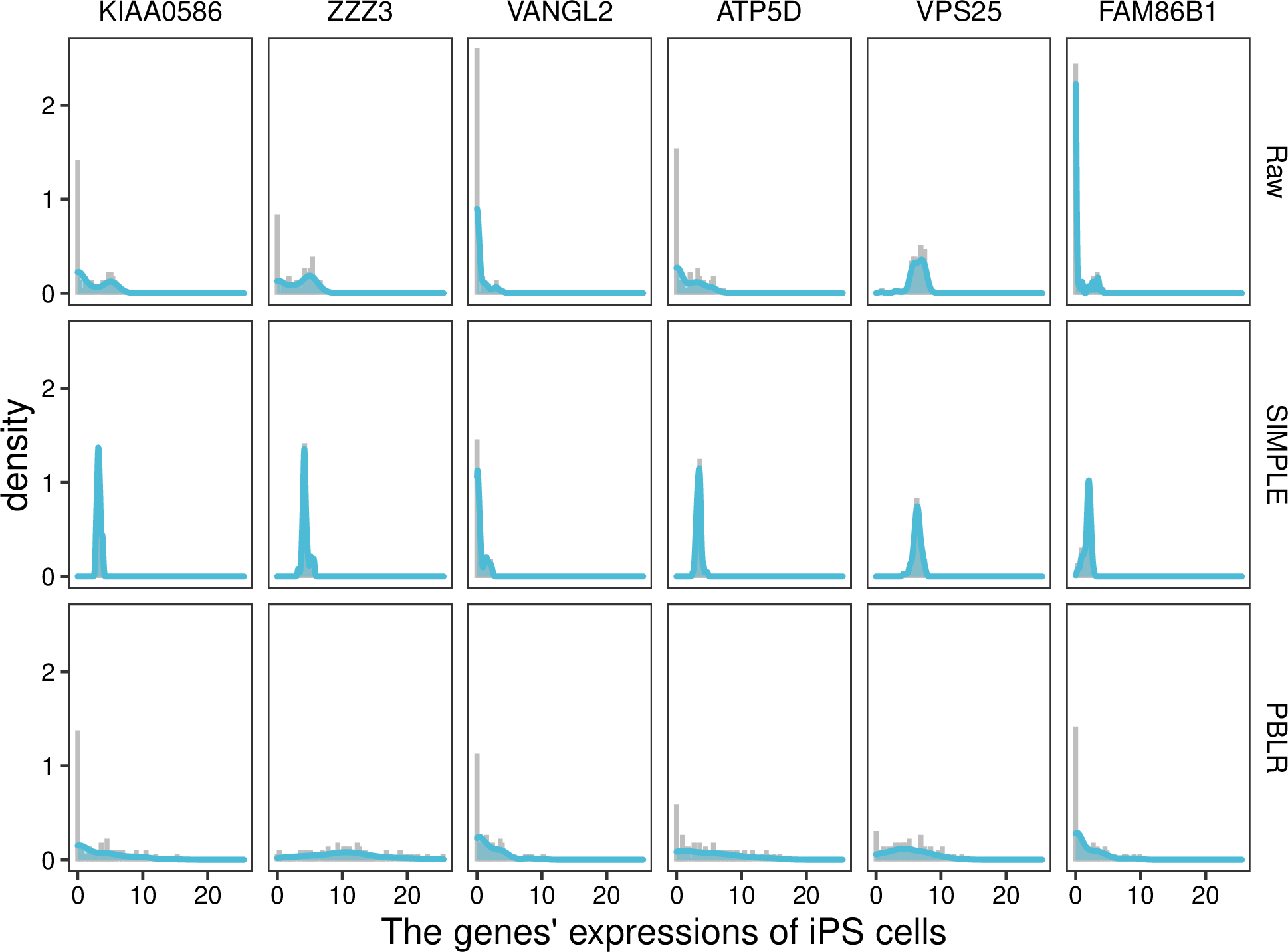

Supplement: S9 Fig — (TIF) [file pcbi.1009118.s010.tif]

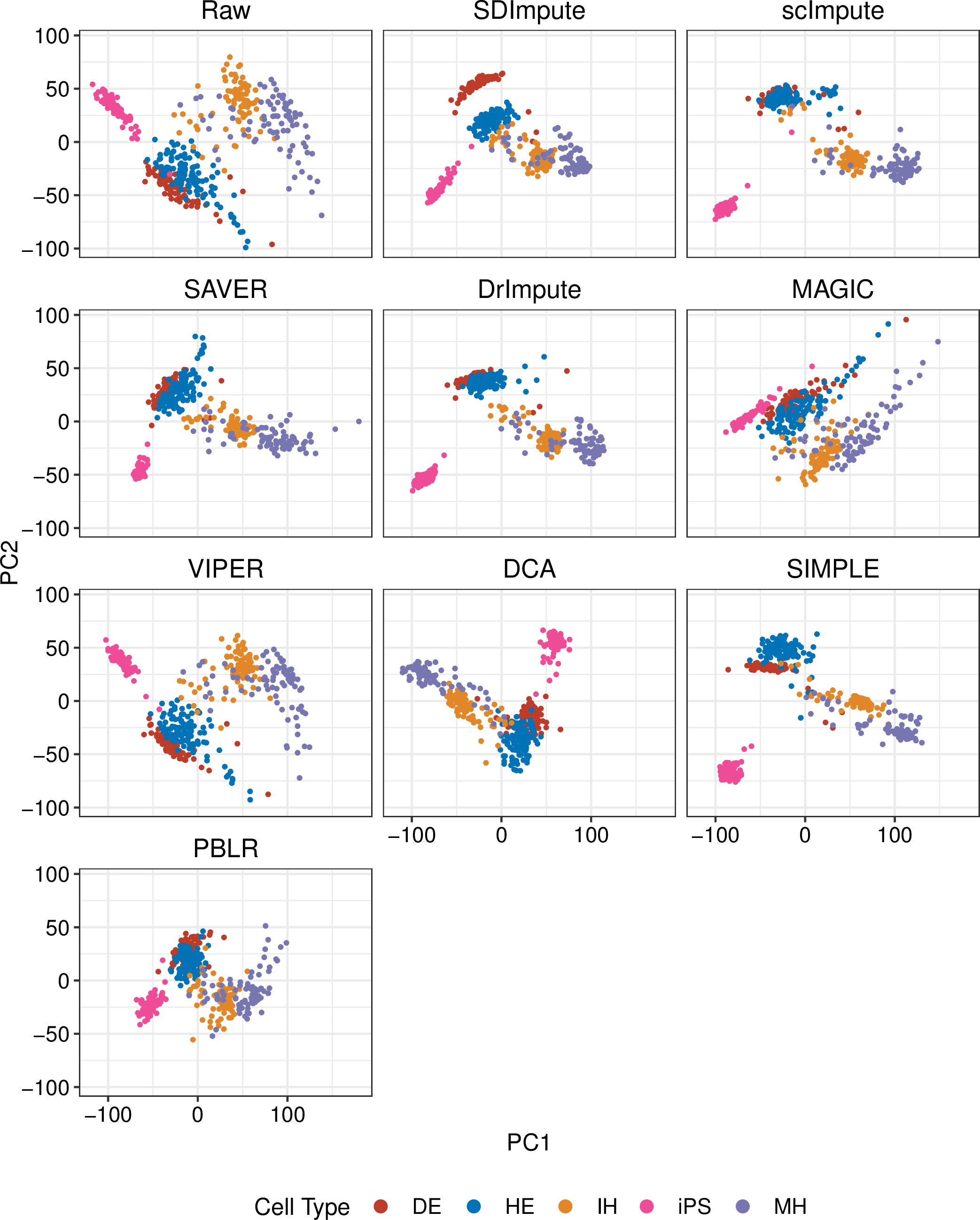

Supplement: S10 Fig — (TIF) [file pcbi.1009118.s011.tif]

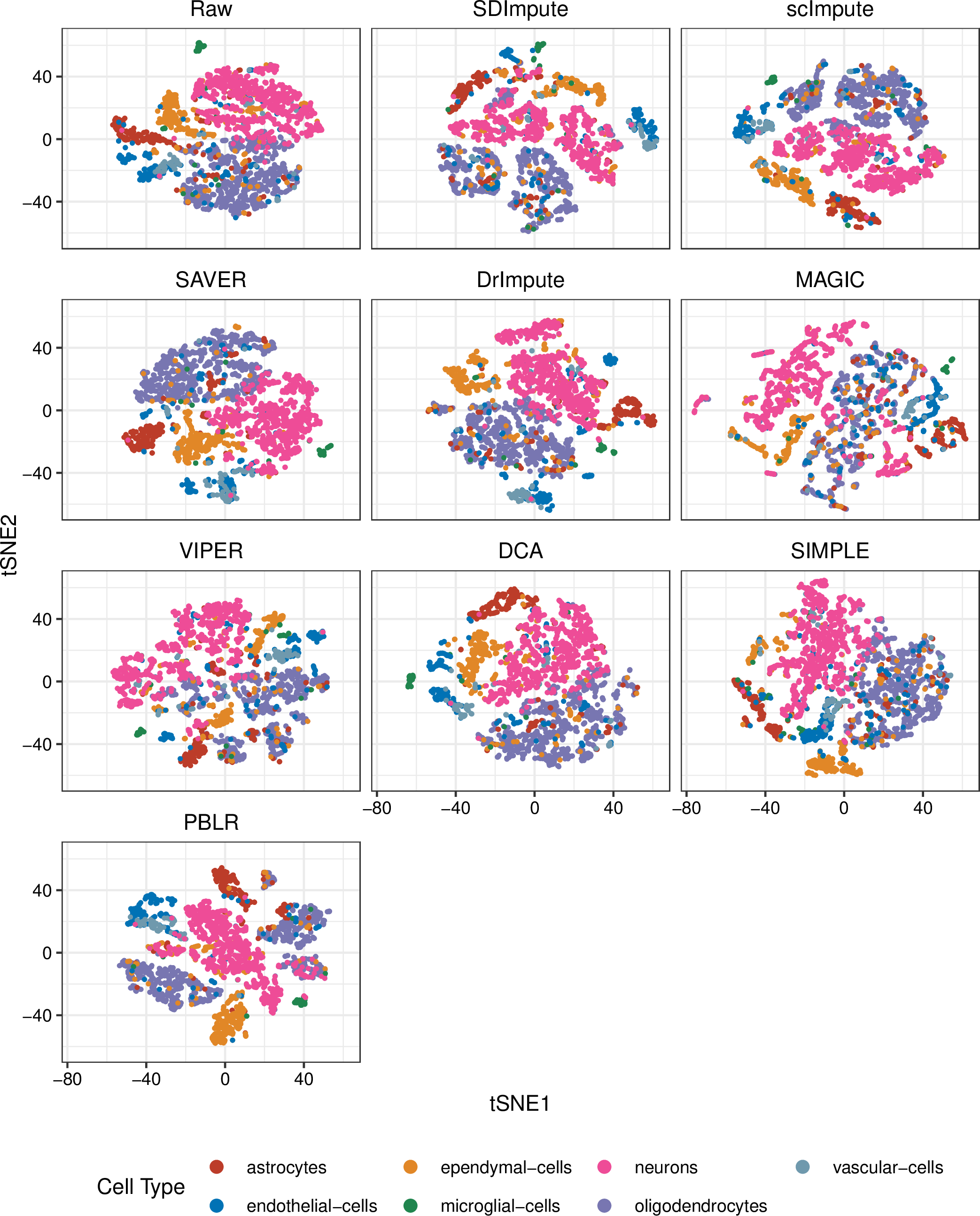

Supplement: S11 Fig — (TIF) [file pcbi.1009118.s012.tif]

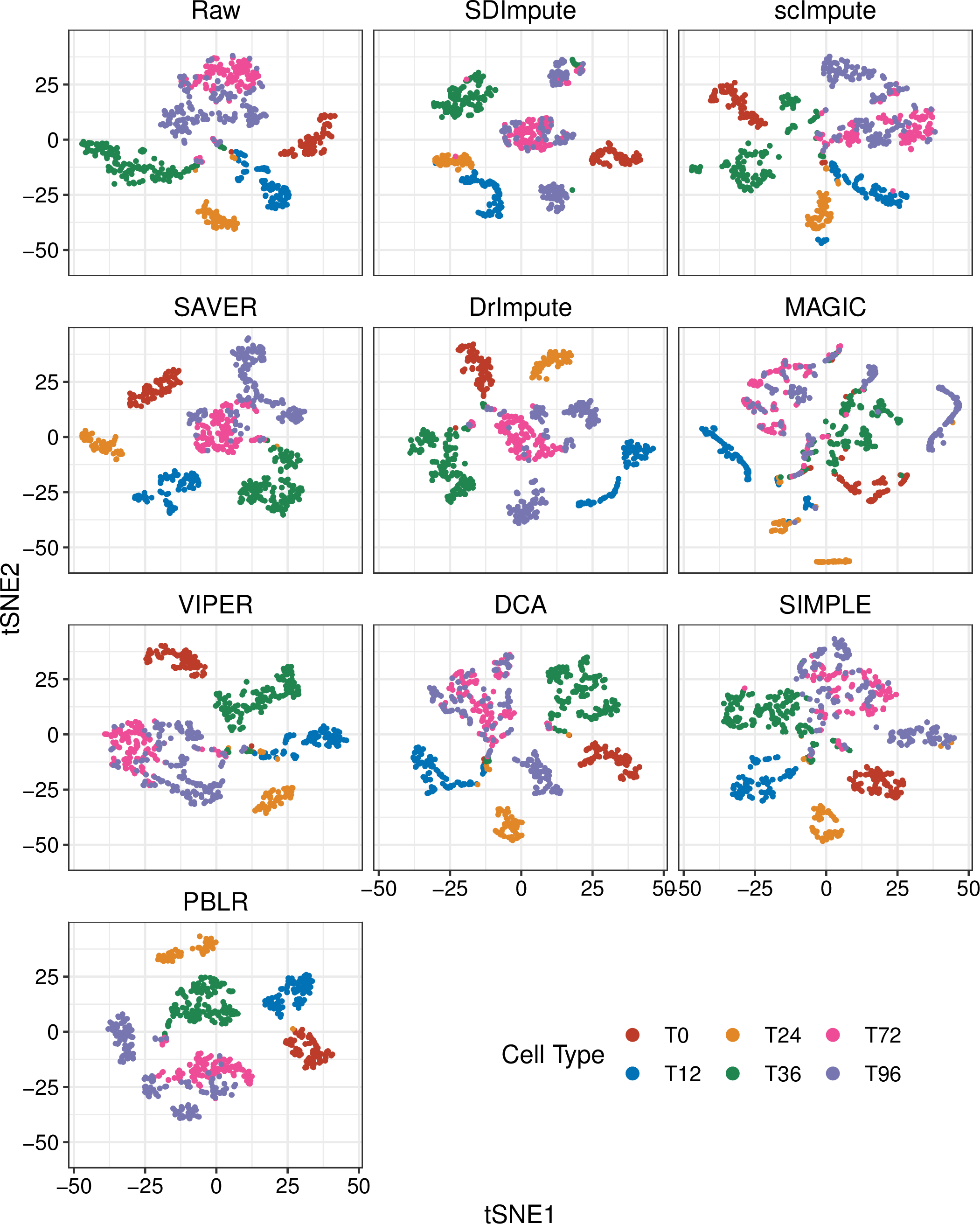

Supplement: S12 Fig — (TIF) [file pcbi.1009118.s013.tif]

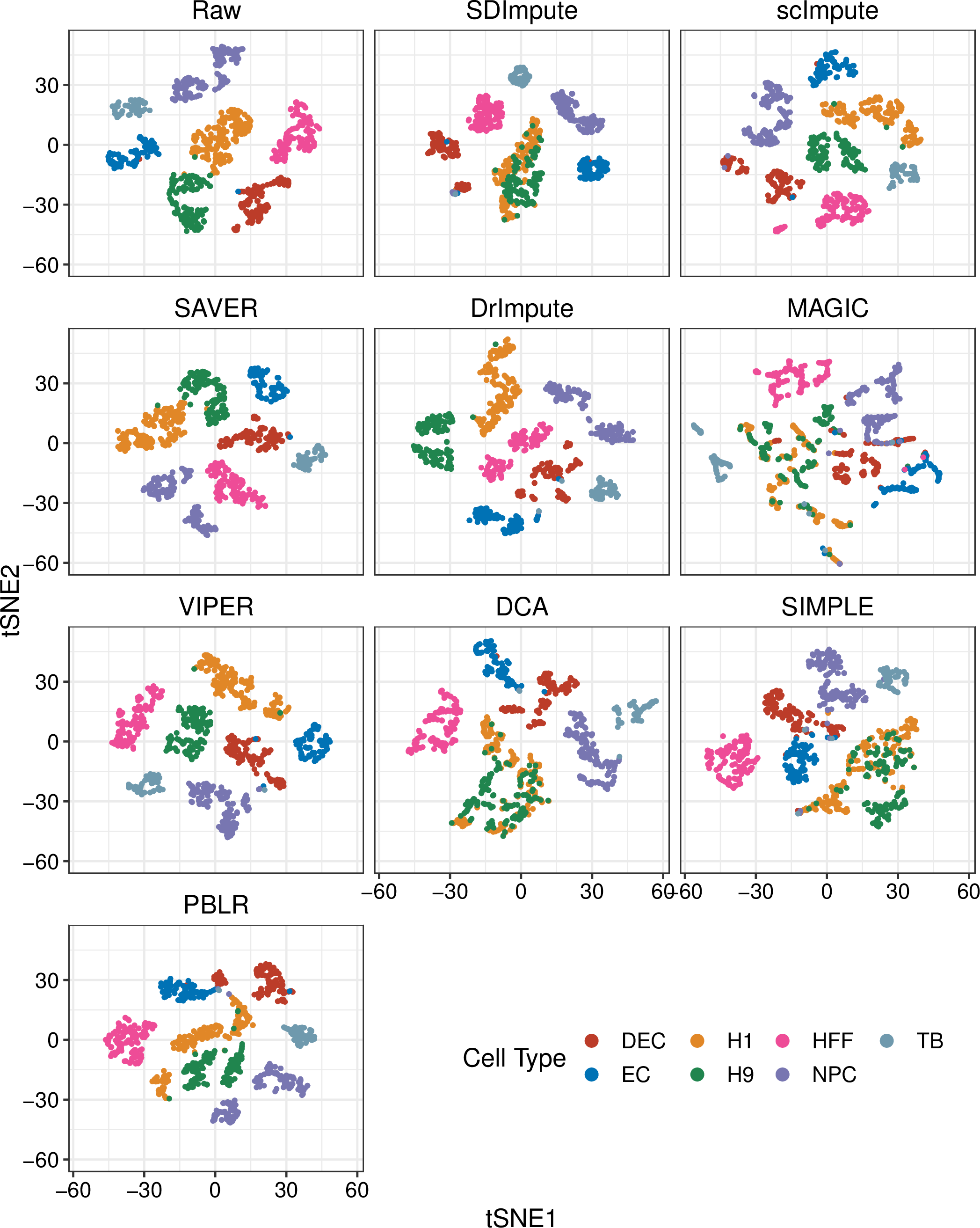

Supplement: S13 Fig — (TIF) [file pcbi.1009118.s014.tif]

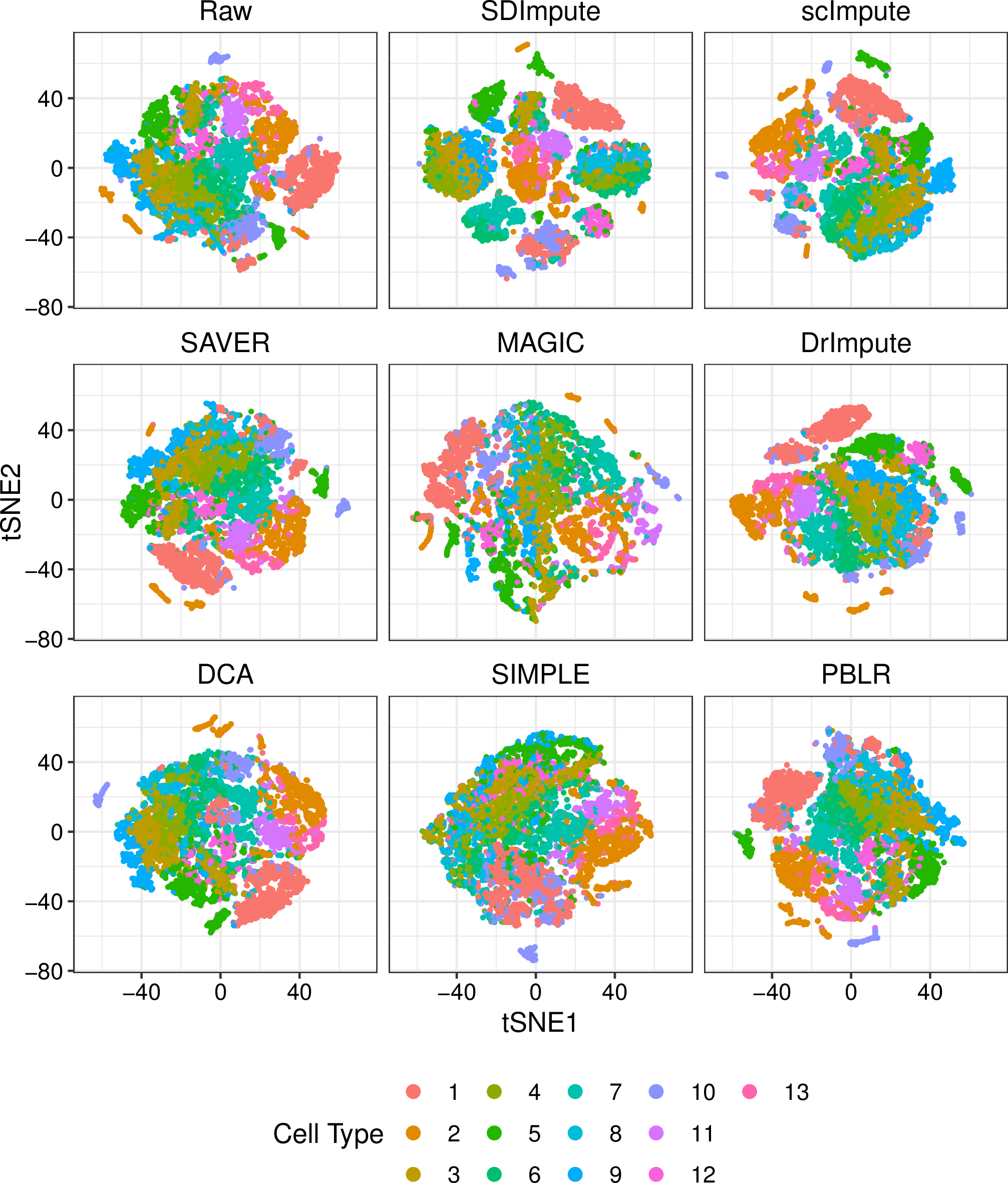

Supplement: S14 Fig — (TIF) [file pcbi.1009118.s015.tif]

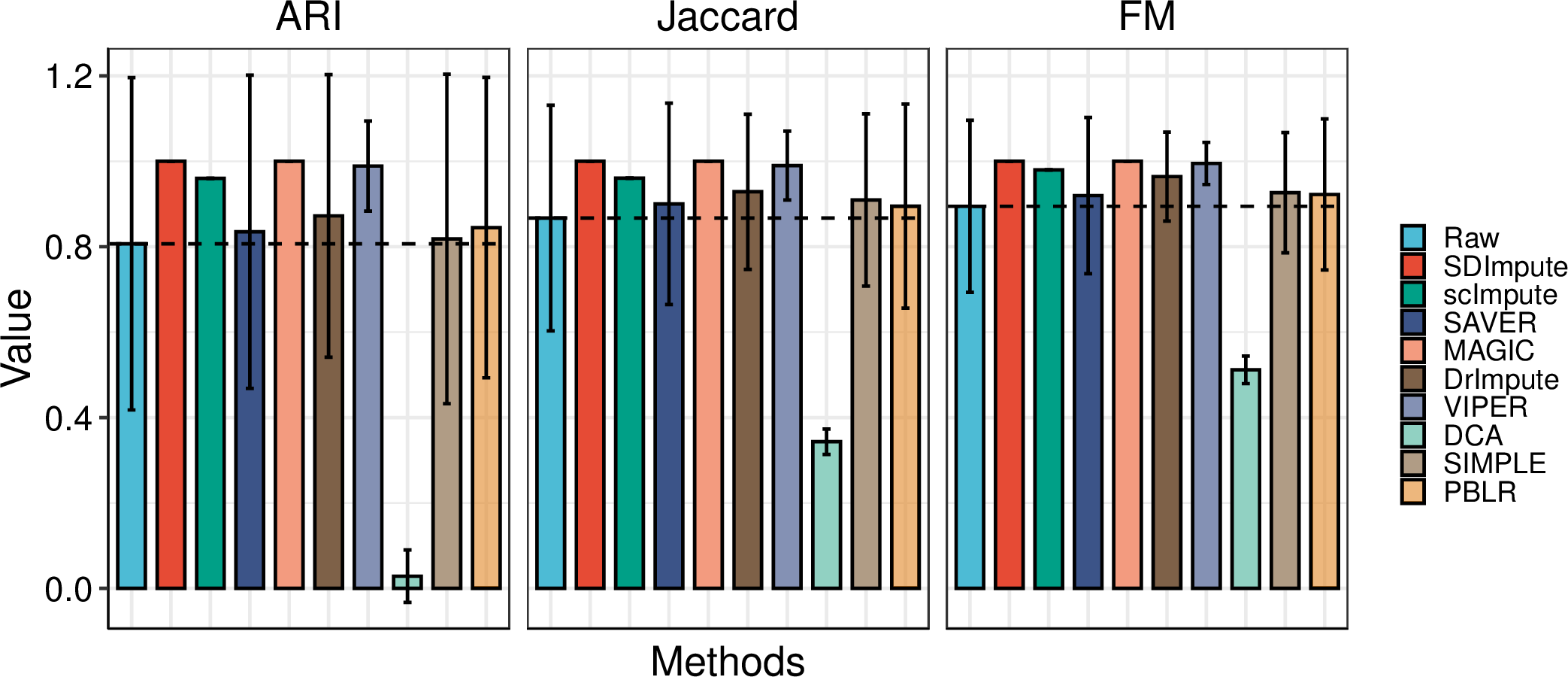

Supplement: S15 Fig — (TIF) [file pcbi.1009118.s016.tif]

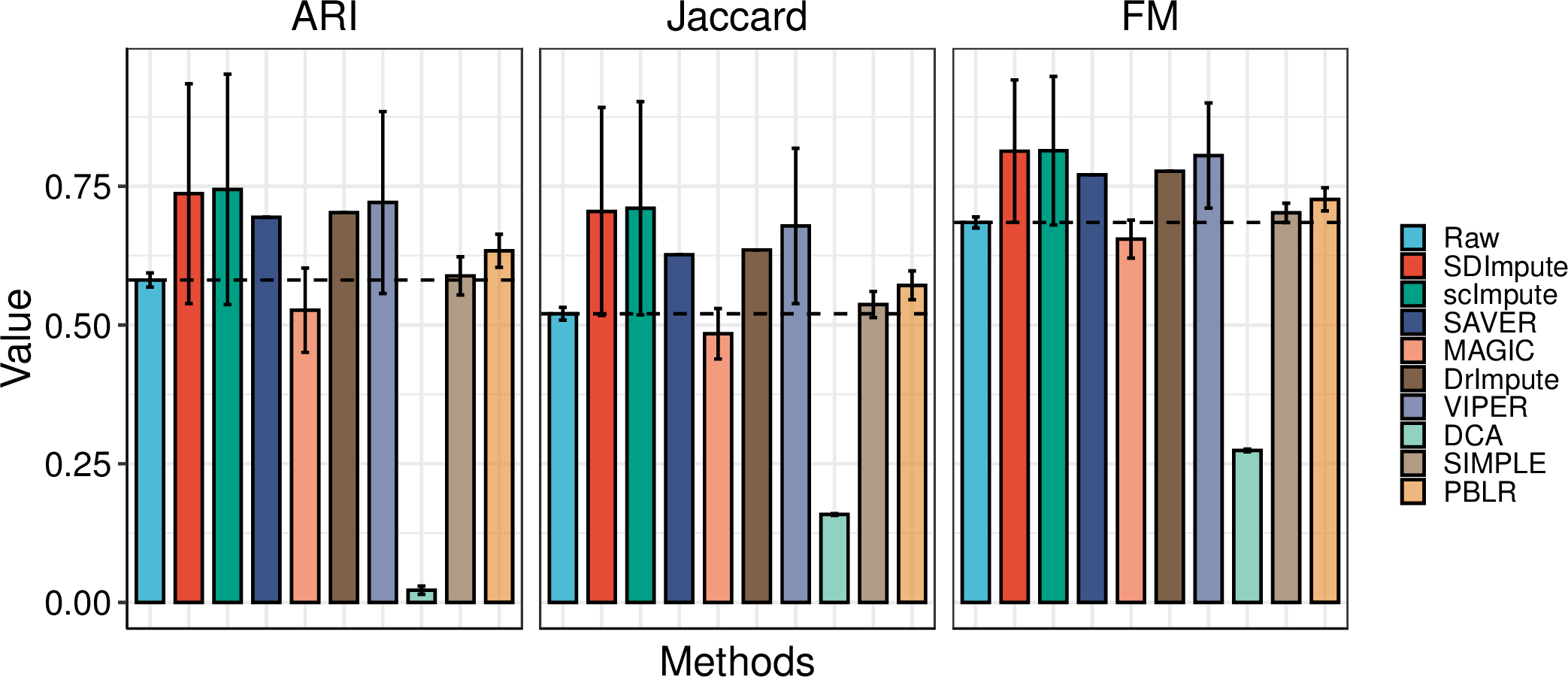

Supplement: S17 Fig — (TIF) [file pcbi.1009118.s018.tif]

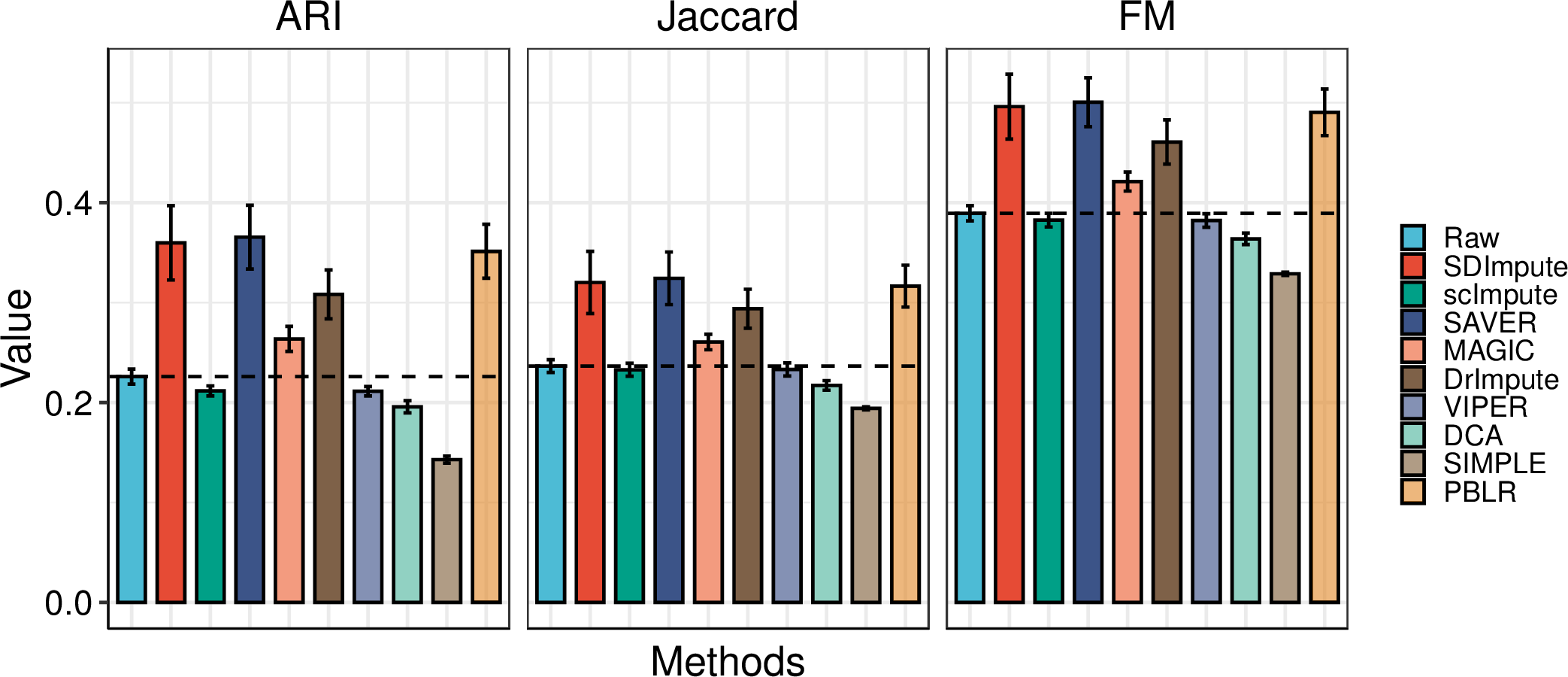

Supplement: S18 Fig — (TIF) [file pcbi.1009118.s019.tif]

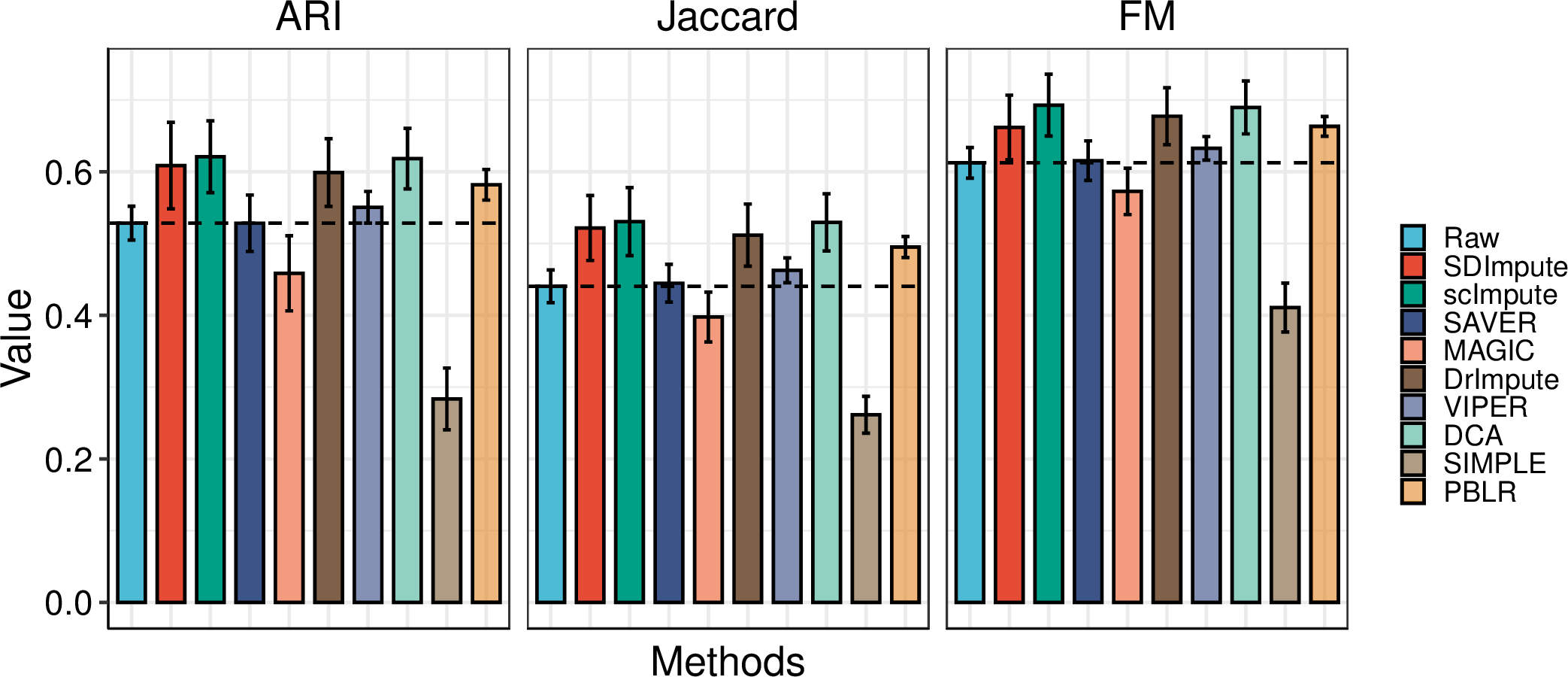

Supplement: S19 Fig — (TIF) [file pcbi.1009118.s020.tif]

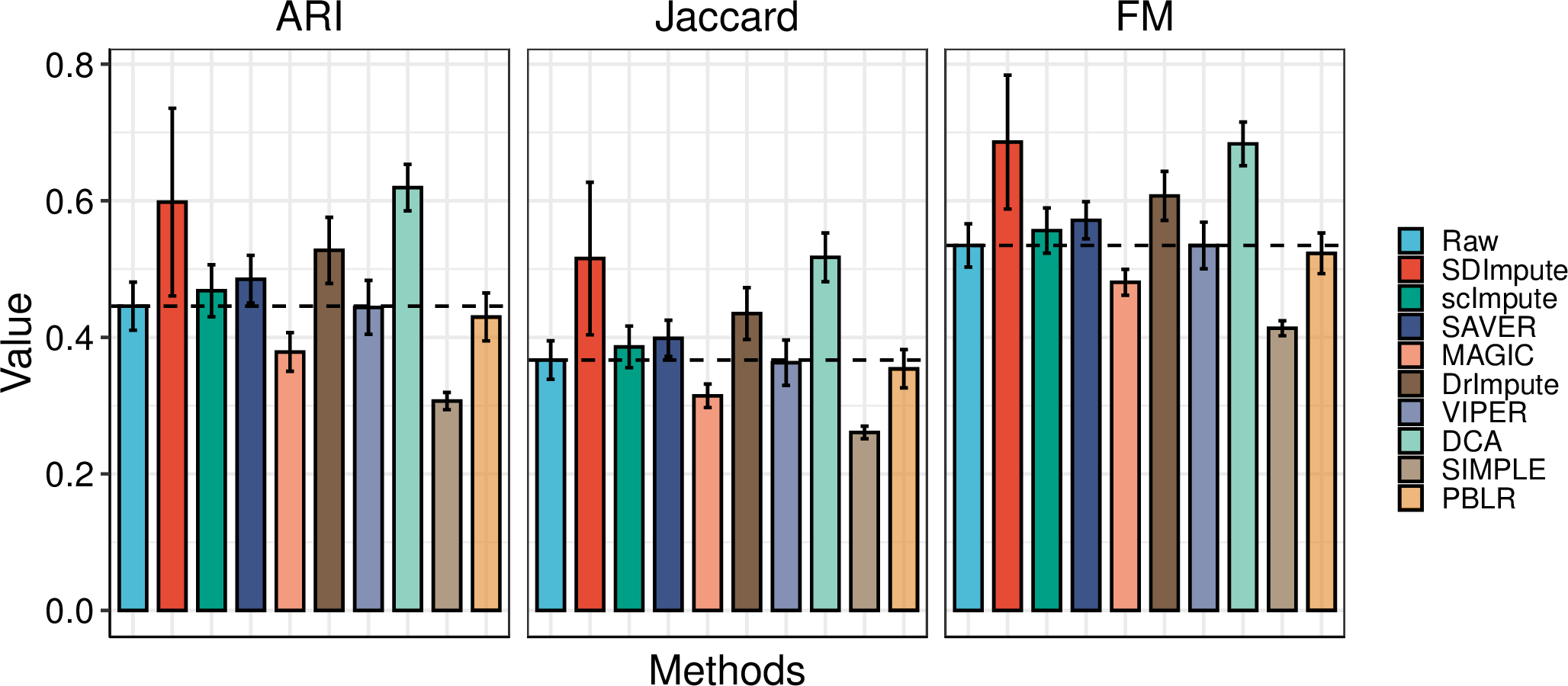

Supplement: S20 Fig — (TIF) [file pcbi.1009118.s021.tif]

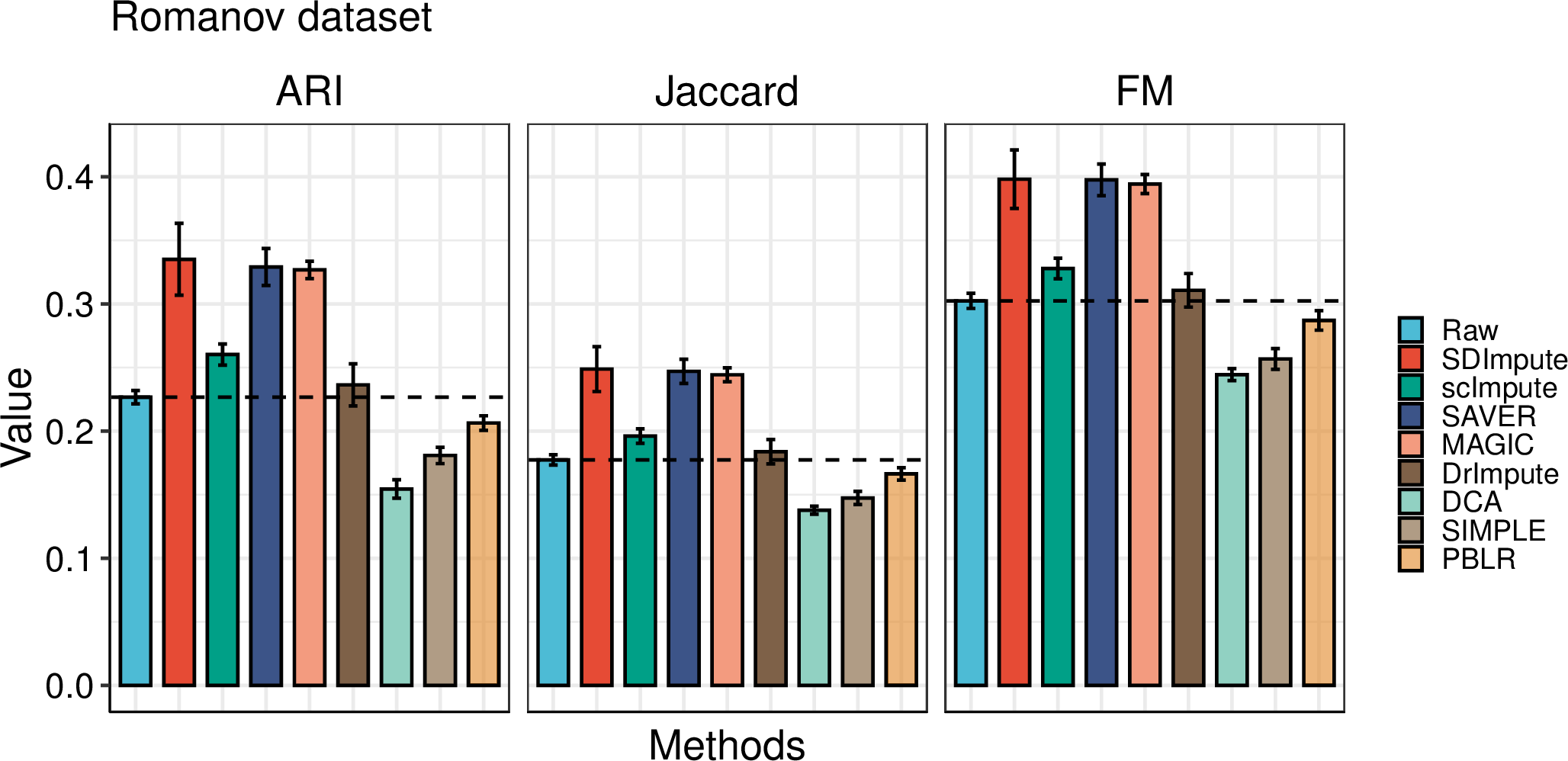

Supplement: S21 Fig — (TIF) [file pcbi.1009118.s022.tif]

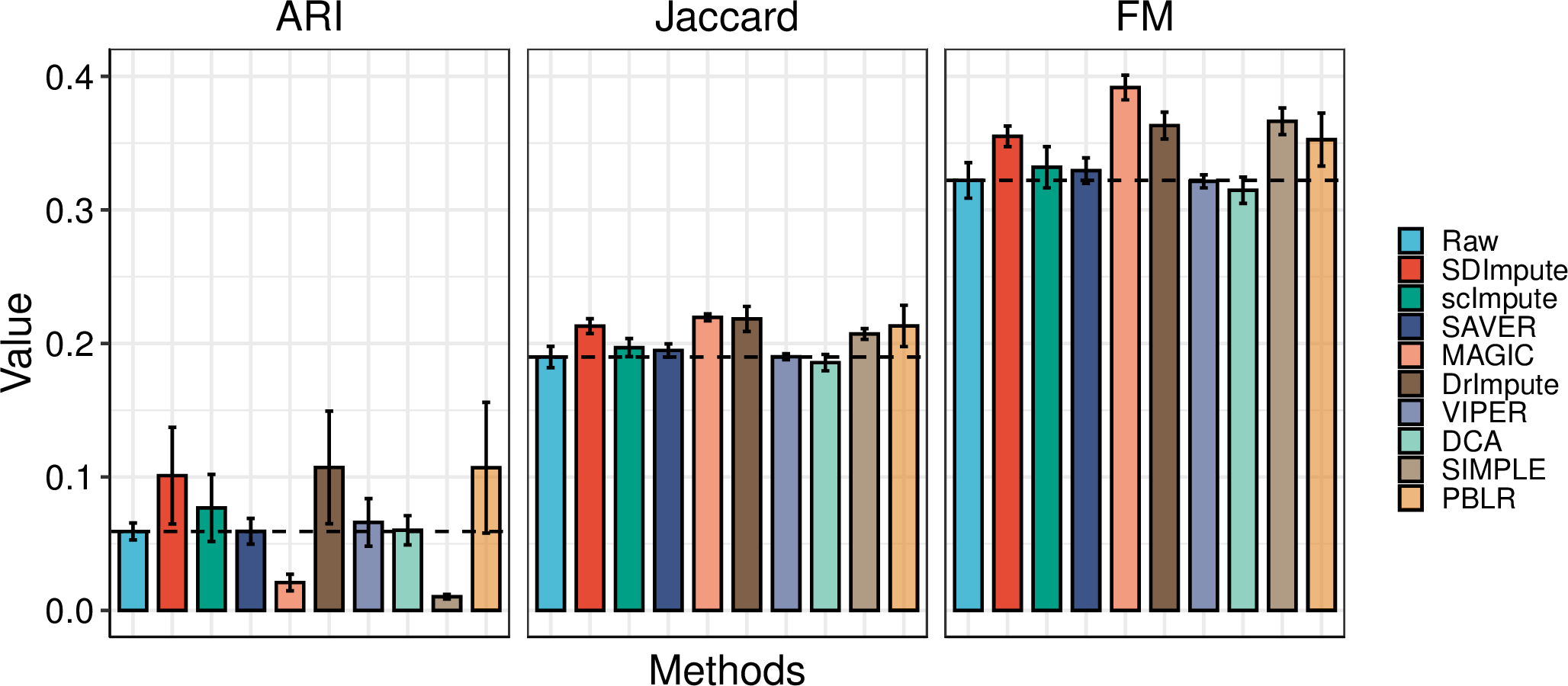

Supplement: S22 Fig — (TIF) [file pcbi.1009118.s023.tif]

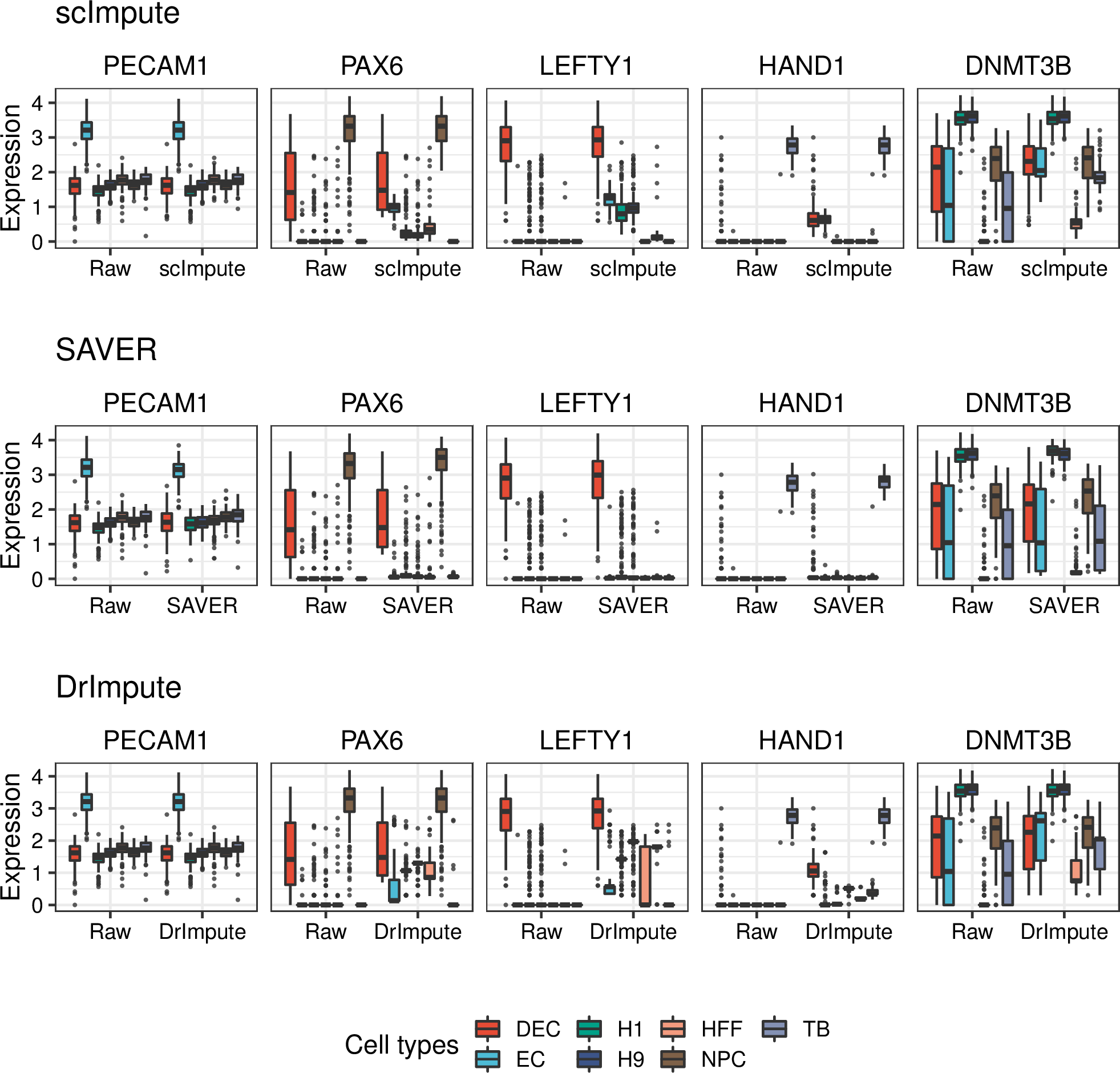

Supplement: S23 Fig — (TIF) [file pcbi.1009118.s024.tif]

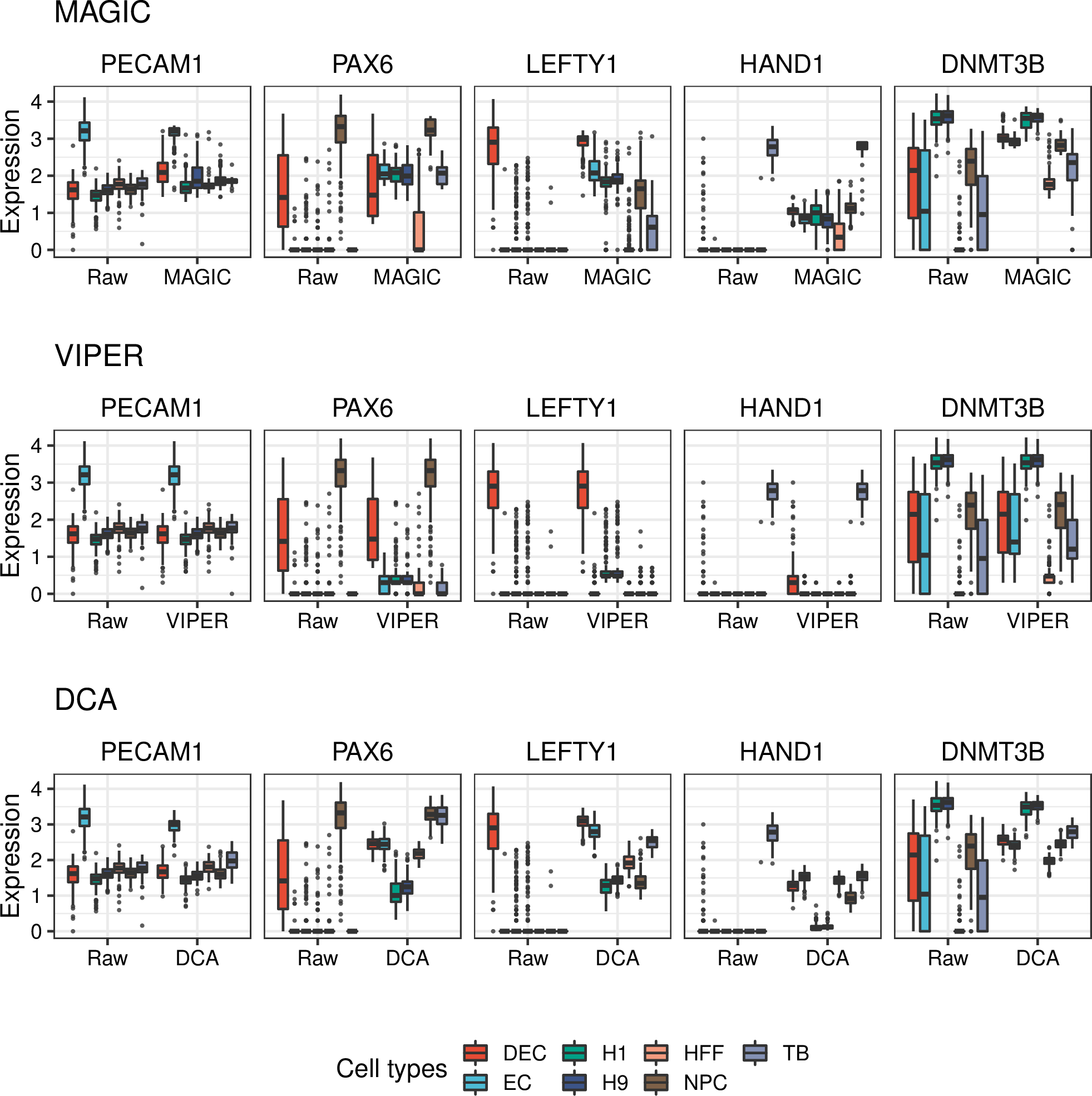

Supplement: S24 Fig — (TIF) [file pcbi.1009118.s025.tif]

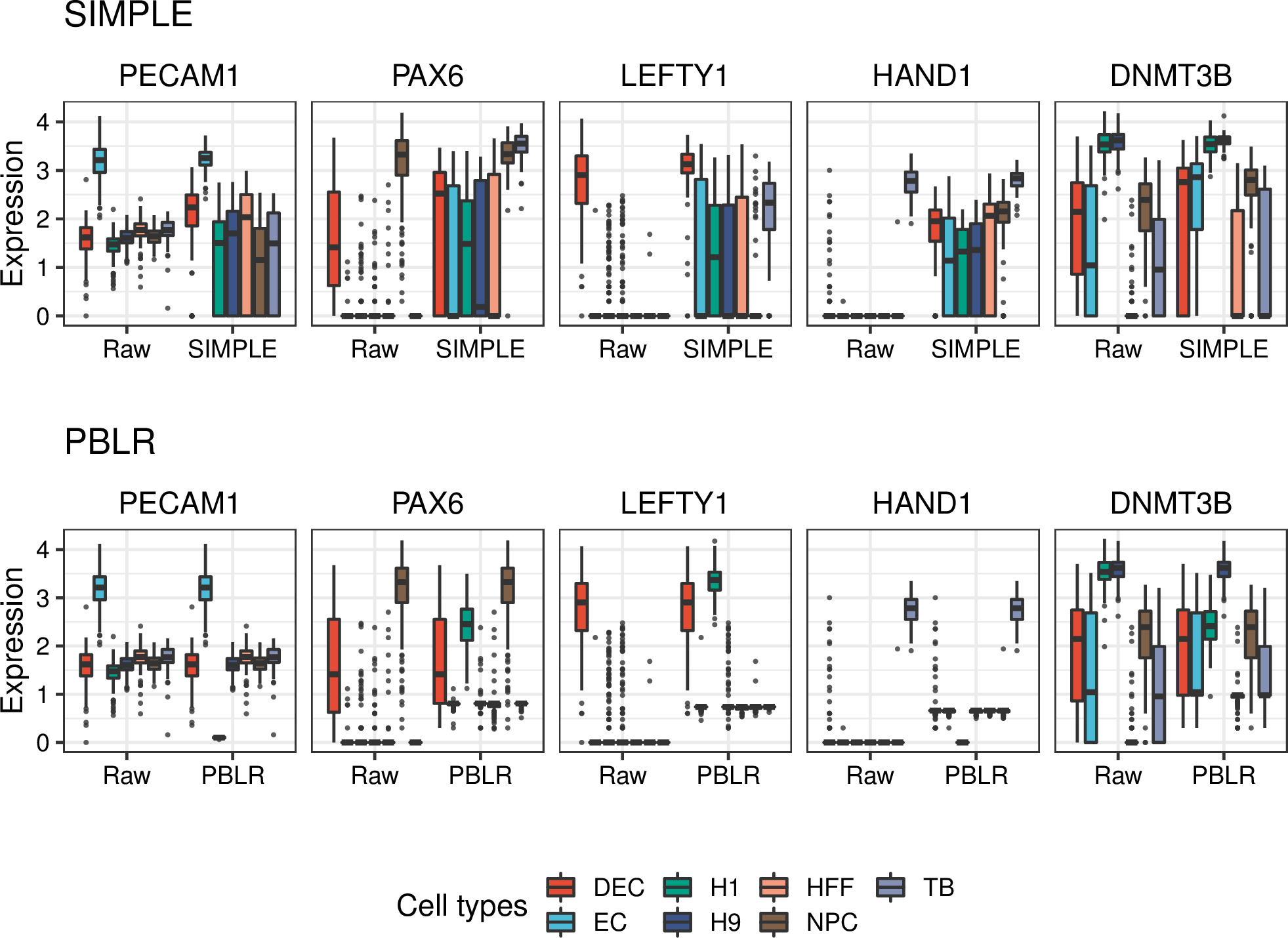

Supplement: S25 Fig — (TIF) [file pcbi.1009118.s026.tif]

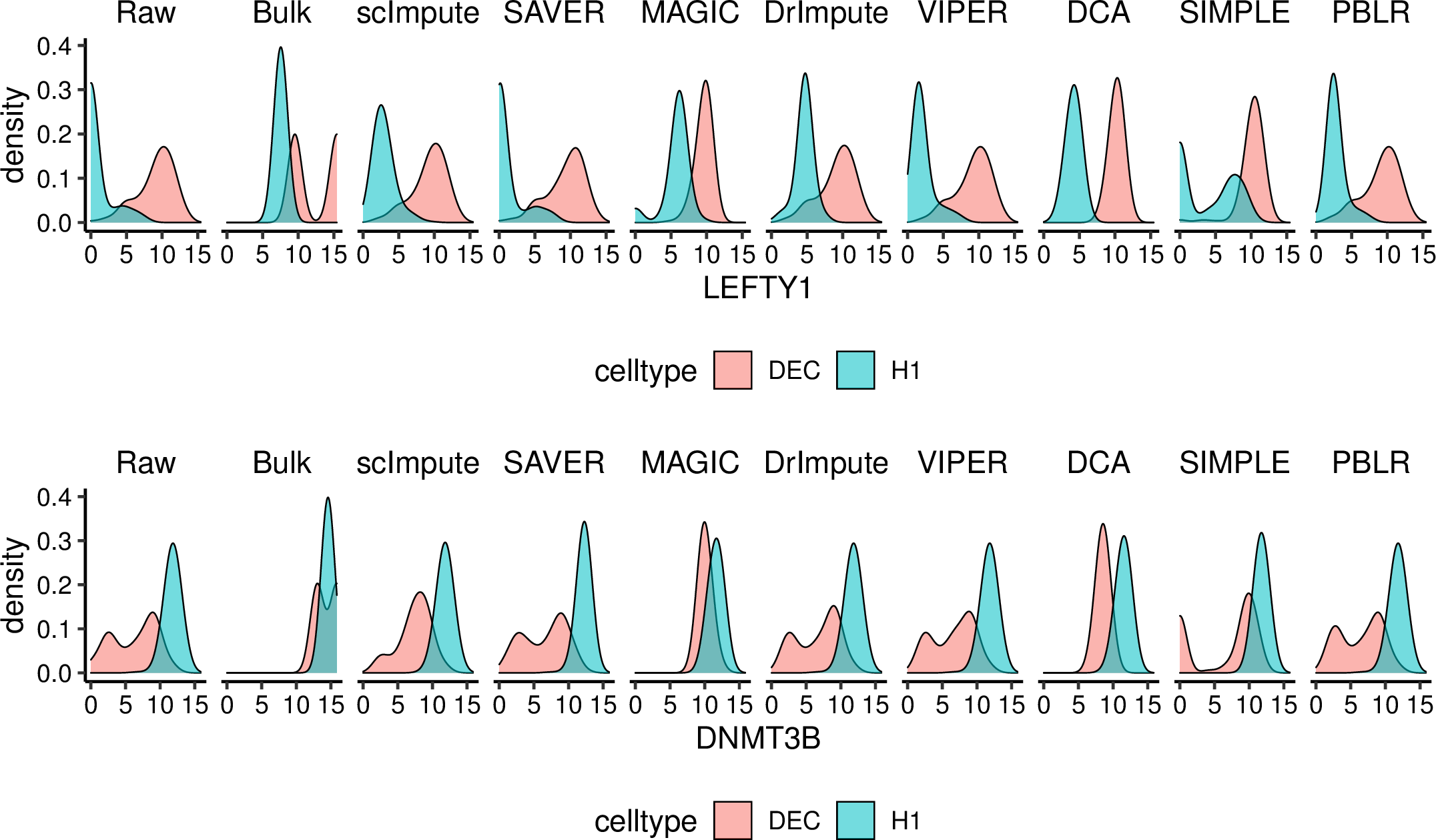

Supplement: S26 Fig — (TIF) [file pcbi.1009118.s027.tif]

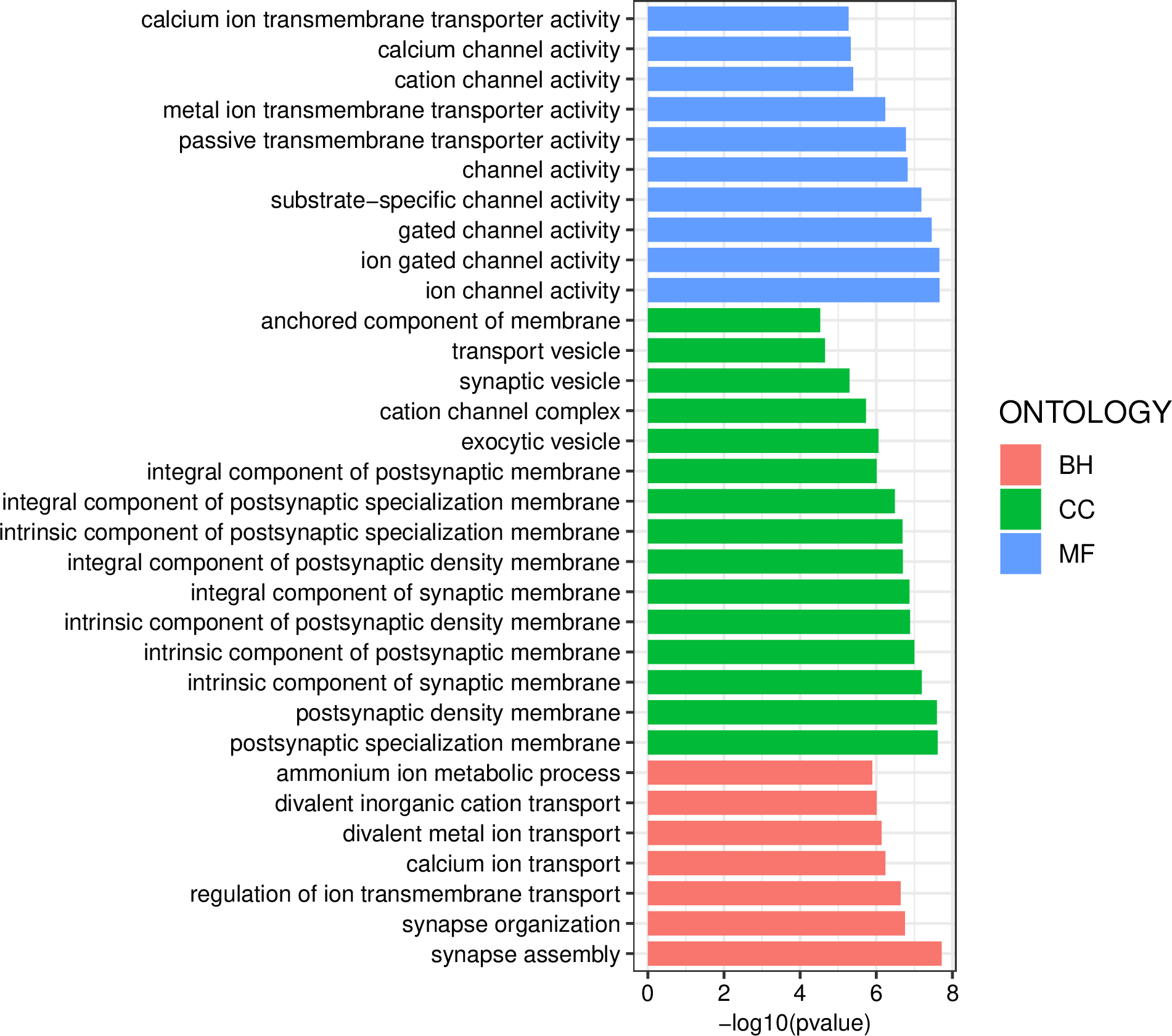

Supplement: S27 Fig — (TIF) [file pcbi.1009118.s028.tif]

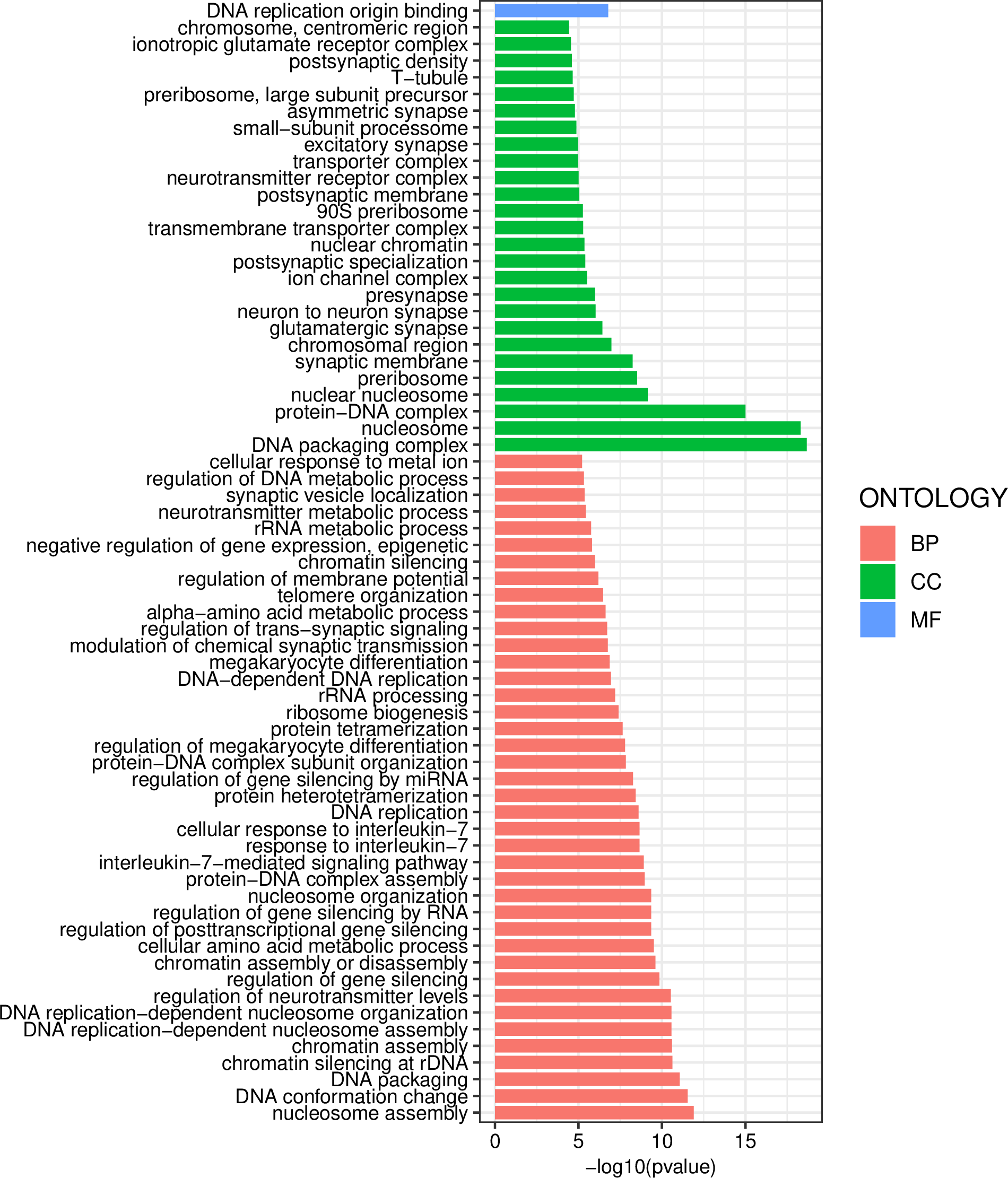

Supplement: S28 Fig — (TIF) [file pcbi.1009118.s029.tif]

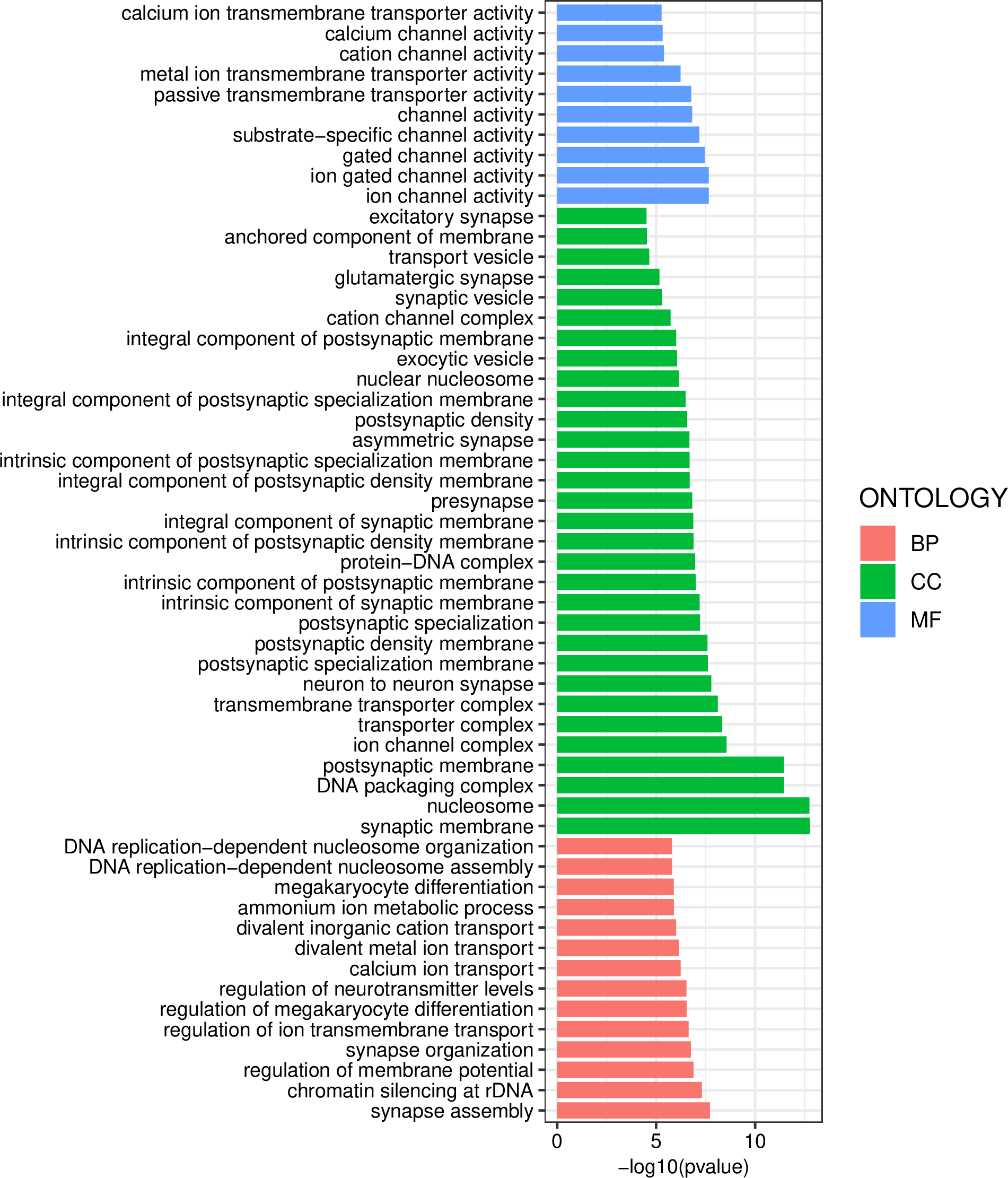

Supplement: S29 Fig — (TIF) [file pcbi.1009118.s030.tif]

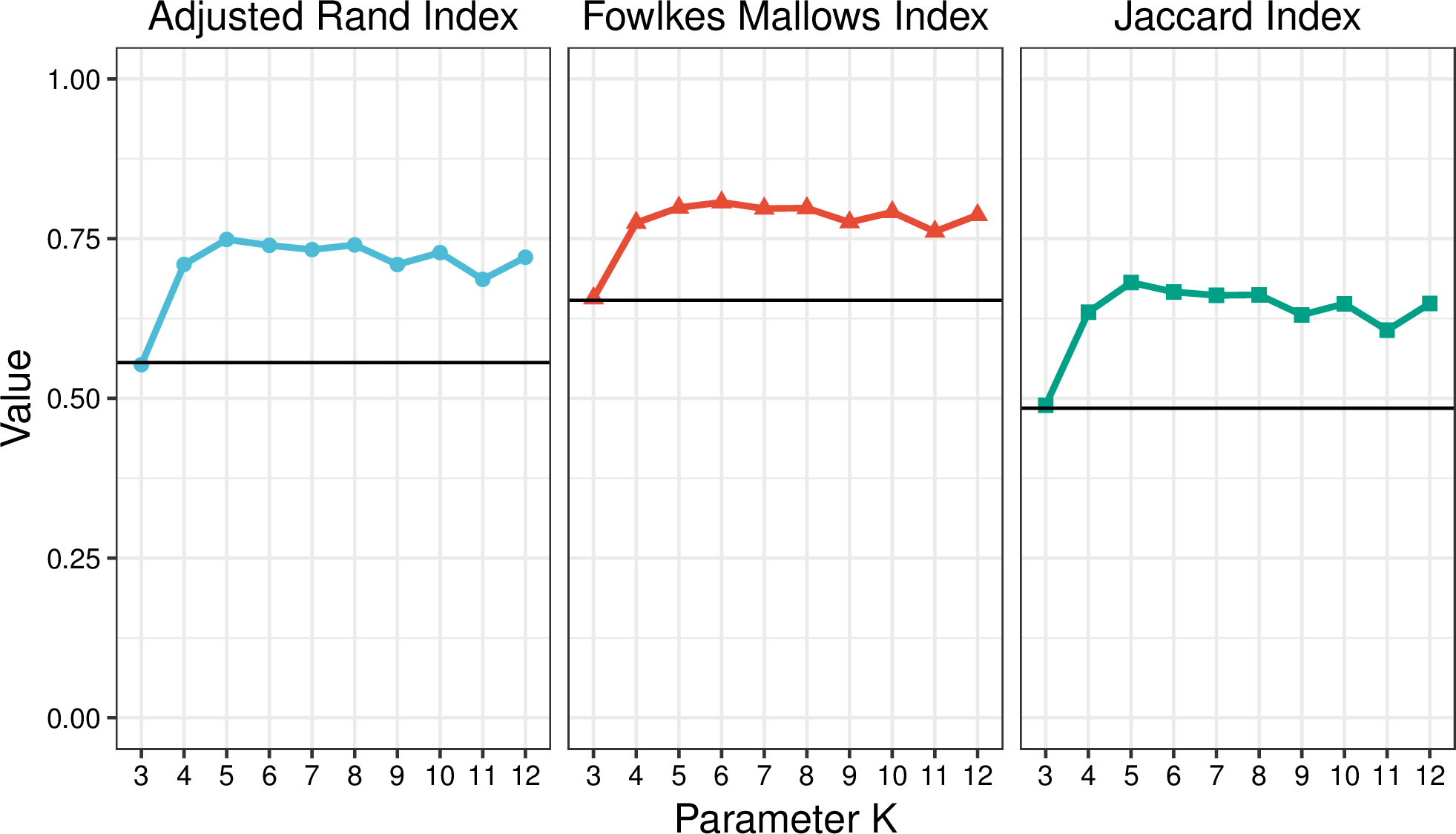

Supplement: S30 Fig — (TIF) [file pcbi.1009118.s031.tif]

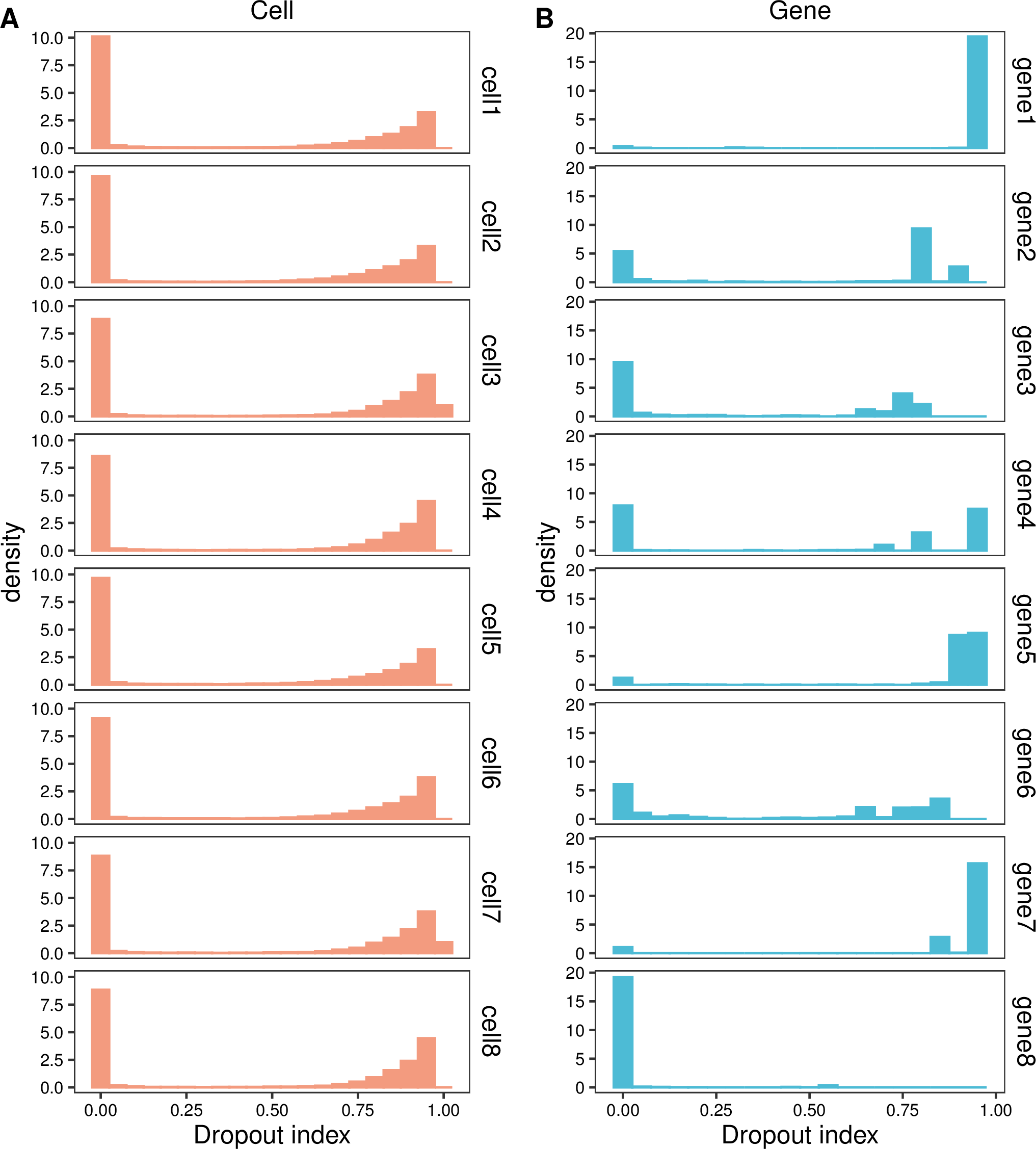

Supplement: S31 Fig — (TIF) [file pcbi.1009118.s032.tif]

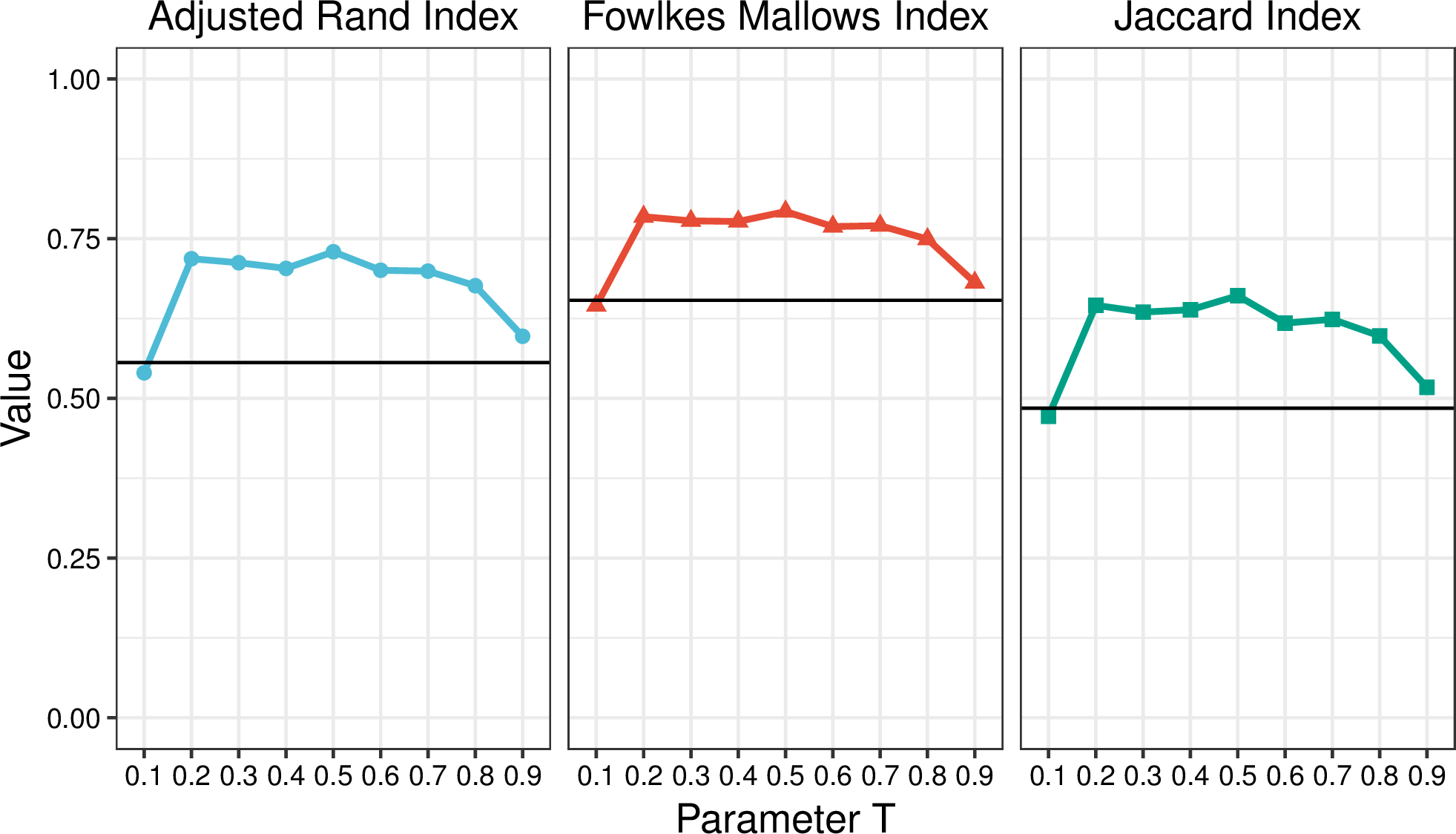

Supplement: S32 Fig — (TIF) [file pcbi.1009118.s033.tif]

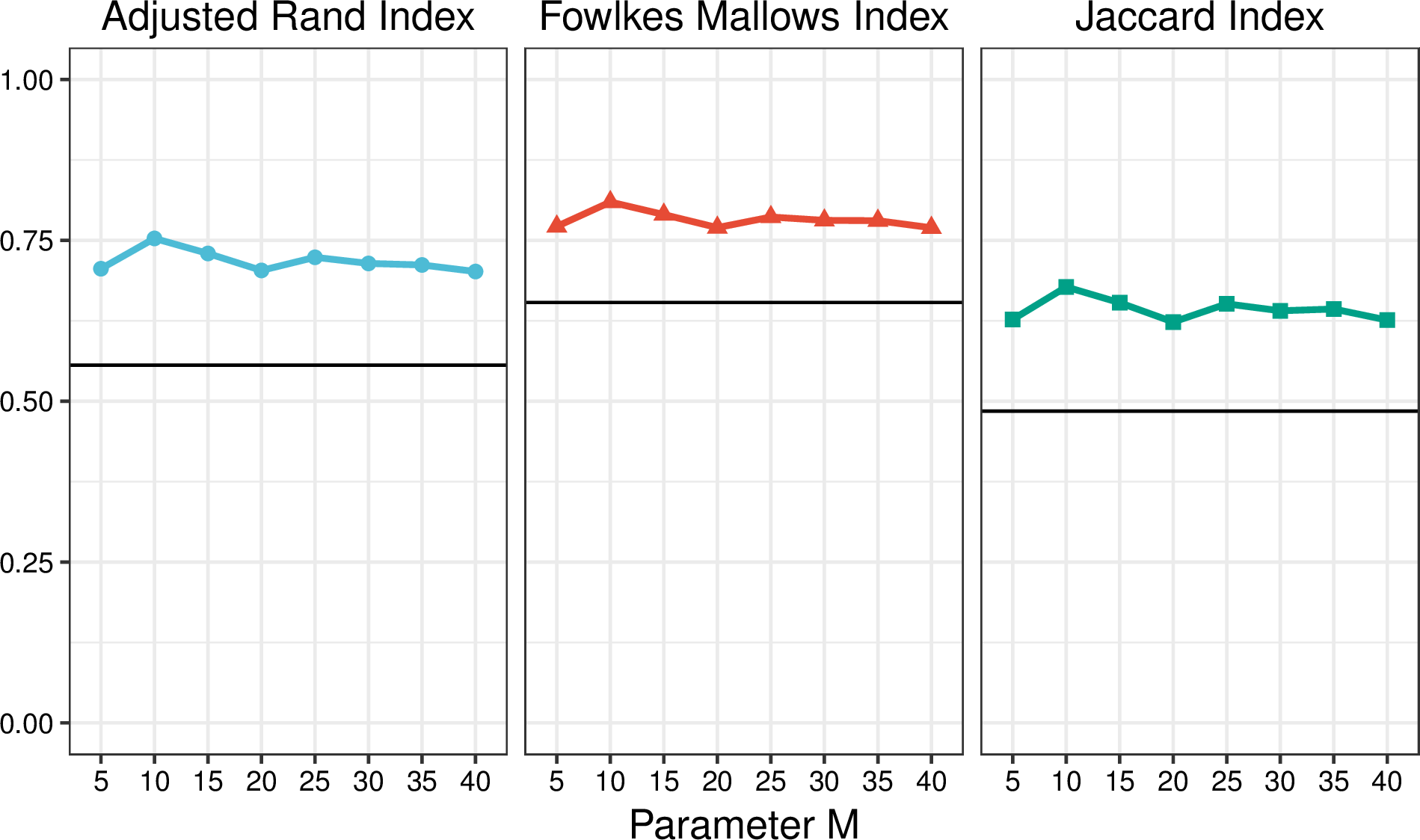

Supplement: S33 Fig — (TIF) [file pcbi.1009118.s034.tif]
